# Genomic variation within the maize stiff‐stalk heterotic germplasm pool

**DOI:** 10.1002/tpg2.20114

**Published:** 2021-07-18

**Authors:** Nolan Bornowski, Kathryn J. Michel, John P. Hamilton, Shujun Ou, Arun S. Seetharam, Jerry Jenkins, Jane Grimwood, Chris Plott, Shengqiang Shu, Jayson Talag, Megan Kennedy, Hope Hundley, Vasanth R. Singan, Kerrie Barry, Chris Daum, Yuko Yoshinaga, Jeremy Schmutz, Candice N. Hirsch, Matthew B. Hufford, Natalia de Leon, Shawn M. Kaeppler, C. Robin Buell

**Affiliations:** ^1^ Dep. of Plant Biology Michigan State Univ. 612 Wilson Road East Lansing MI 48824 USA; ^2^ Dep. of Agronomy Univ. of Wisconsin – Madison 1575 Linden Drive Madison WI 53706 USA; ^3^ Dep. of Ecology, Evolution, and Organismal Biology Iowa State Univ. 2200 Osborn Drive Ames IA 50011 USA; ^4^ HudsonAlpha Institute for Biotechnology 601 Genome Way Northwest Huntsville AL 35806 USA; ^5^ U.S. Dep. of Energy Joint Genome Institute Lawrence Berkeley National Laboratory 1 Cyclotron Road Berkeley CA 94720 USA; ^6^ Arizona Genomics Institute School of Plant Sciences Univ. of Arizona 1657 E Helen Street Tucson AZ 85721 USA; ^7^ Dep. of Agronomy and Plant Genetics Univ. of Minnesota 1991 Upper Buford Circle Saint Paul MN 55108 USA; ^8^ Dep. of Energy, Great Lakes Bioenergy Research Center Univ. of Wisconsin – Madison 1575 Linden Drive Madison WI 53706 USA; ^9^ Wisconsin Crop Innovation Center Univ. of Wisconsin – Madison 8520 University Green Middleton WI 53562 USA; ^10^ Dep. of Energy, Great Lakes Bioenergy Research Center Michigan State Univ. 612 Wilson Road East Lansing MI 48824 USA

## Abstract

The stiff‐stalk heterotic group in Maize (*Zea mays* L.) is an important source of inbreds used in U.S. commercial hybrid production. Founder inbreds B14, B37, B73, and, to a lesser extent, B84, are found in the pedigrees of a majority of commercial seed parent inbred lines. We created high‐quality genome assemblies of B84 and four expired Plant Variety Protection (ex‐PVP) lines LH145 representing B14, NKH8431 of mixed descent, PHB47 representing B37, and PHJ40, which is a Pioneer Hi‐Bred International (PHI) early stiff‐stalk type. Sequence was generated using long‐read sequencing achieving highly contiguous assemblies of 2.13–2.18 Gbp with N50 scaffold lengths >200 Mbp. Inbred‐specific gene annotations were generated using a core five‐tissue gene expression atlas, whereas transposable element (TE) annotation was conducted using de novo and homology‐directed methodologies. Compared with the reference inbred B73, synteny analyses revealed extensive collinearity across the five stiff‐stalk genomes, although unique components of the maize pangenome were detected. Comparison of this set of stiff‐stalk inbreds with the original Iowa Stiff Stalk Synthetic breeding population revealed that these inbreds represent only a proportion of variation in the original stiff‐stalk pool and there are highly conserved haplotypes in released public and ex‐Plant Variety Protection inbreds. Despite the reduction in variation from the original stiff‐stalk population, substantial genetic and genomic variation was identified supporting the potential for continued breeding success in this pool. The assemblies described here represent stiff‐stalk inbreds that have historical and commercial relevance and provide further insight into the emerging maize pangenome.

## Core Ideas

- Genome assemblies and annotations of five ex‐PVP stiff‐stalk inbred lines were generated.
- Stiff‐stalk pangenome has limited diversity compared with the overall maize pangenome.
- Stiff‐stalk lines contain distinct haplotypes that have been conserved in breeding germplasm.

**Abbreviations**
BSSS: Iowa Stiff Stalk Synthetic
CDS: coding sequence
EDTA: extensive de‐novo transposable element annotator
ex‐PVP: expired Plant Variety Protection
fl‐LTR: full‐length long‐terminal repeat
IBS: identity by state
INDEL: insertion–deletion
LAI: long‐terminal repeat assembly index
LTR: long‐terminal repeat
MTEC: Maize TE Consortium
PAV: presence–absence variant
PHI: Pioneer Hi‐Bred International
PVP: Plant Variety Protection
RNA‐seq: RNA sequencing
SNP: single nucleotide polymorphism
SV: structural variant
TE: transposable element
WGS: whole‐genome shotgun

## 1 INTRODUCTION

Maize (*Zea mays* L.) production is vital to American agriculture and the global food supply, and significant heterosis, or the superior performance of a hybrid progeny over its inbred parents, exists in maize. Heterosis generated from the cross of two unrelated inbreds from opposing heterotic groups has supported immense yield gains since the introduction of the hybrid cross in the early 20th century. Modern maize breeding relies on several key heterotic groups and subgroups (White et al., 2020) with new inbreds generated within heterotic groups and hybrids generated from crosses between heterotic groups. Heterotic patterns did not arise out of a conscious decision to create them but rather as a necessity for organization within breeding programs (Tracy & Chandler, 2006). Initial pools were made arbitrarily by some programs, while others attempted to group related lines together. For example, Pioneer Hi‐Bred International (PHI) made efforts to gather good seed parents in one group and good pollen parents in the other (Tracy & Chandler, 2006). Over time, the contrasting pools genetically diverged, as evidenced by a study of inbreds used from the early 1930s to 2001 at PHI (Duvick, 2005). Using simple sequence repeat markers and multidimensional scaling, the author demonstrated that inbreds used in the “pre‐heterotic” era do not cluster in a discernible pattern, while advanced inbreds classified as either stiff‐stalk or non‐stiff‐stalk form two distinct groups (Duvick, 2005). This allelic diversity led to the great success of the heterotic pattern breeding method as alleles are fixed for contrasting allelic states between heterotic pools, contributing to additive‐by‐additive epistasis and repulsion phase linkages that create pseudo‐overdominance (Graham et al., 1997; Larièpe et al., 2012).

Corporations, individuals, and public institutions can protect inbred lines with Plant Variety Protection (PVP) certificates, which allow the breeder or organization sole ownership over sales of the hybrid progeny for 20 yr in the case of maize, at which point, the certificates expire (Plant Variety Protection Act, 1970). The rapidly increasing number of expired PVP (ex‐PVP) certificates gives public entities the unique opportunity to characterize the pedigrees, genetic diversity, and phenotypic characteristics of elite ex‐PVP lines that originate from a diverse group of breeding programs and contain the parent inbreds that have supported the hybrid maize industry. Several heterotic groups have emerged over the last few decades, which can be studied as the PVP certificates on inbreds expire and biological materials become freely available. Broadly, the major groups are the stiff‐stalk, Iodent, and non‐stiff‐stalk heterotic pools. The Iodent group as represented in ex‐PVP inbreds was founded by PH207 (Hirsch et al., 2016) and has the most limited genetic diversity. The stiff‐stalk heterotic pool, as described below, is also more limited in diversity than the non‐stiff‐stalk pool, which comprises most other lines not grouping as Iodent or stiff‐stalk. Each group has a unique history of selection and development.

The stiff‐stalk heterotic group originated from the Iowa Stiff Stalk Synthetic (BSSS) developed by Dr. George Sprague at Iowa State University in the 1930s. The BSSS is composed of 16 inbred lines, primarily of Reid Yellow Dent heritage, and underwent several cycles of recurrent selection (Troyer, 1999). The population has yielded several key founder inbreds, including B14, released by Sprague in 1953; B37, released by Sprague in 1958; and B73, released by Dr. Wilbert Russell in 1972 (Troyer, 1999). Related samples of the population were used in other public breeding programs and resulted in release of inbreds including N7A and N28, for example (npgsweb.ars‐grin.gov). Inbred B14 was a first cycle selection from the BSSS and was chosen for its superior yield and stalk and root strength and was used heavily in the development of inbreds adapted for early maturity zones such as the northern United States, Canada, and Europe including A632 and A634 (Troyer, 1999). Inbred B37 was also released from the first cycle of selection of the BSSS because of its positive contributions to hybrid yield and agronomic quality but faced issues of low pollen shed and a protracted anthesis–silking interval (Troyer, 1999). Inbred B73 was chosen from cycle five for its high yield in test‐cross hybrids (Troyer, 1999). The B73 × Mo17 hybrid was incredibly popular and grown across the American Corn Belt during the 1970s, and B73 would later serve as the first representative reference assembly of maize (Schnable et al., 2009). Goodman (1990) estimated that perhaps 70% of the hybrid commercial germplasm in 1990 relied on close relatives of just six inbreds including Lancaster lines C103, Mo17, and Oh43, and stiff‐stalk lines B73, B37, and A632 (a B14 derivative) as the seed parent. These three stiff‐stalk inbreds were heavily used by private seed companies as foundational inbreds within their breeding programs and were valued for their superior seed parent characteristics. Thus, the stiff‐stalk heterotic group was, and is, vital for North American hybrid maize production.

The first maize reference genome assembly was generated from B73 (Schnable et al., 2009). Several maize genome assemblies have since been published including tropical lines CML247 and SK (Lu et al., 2015; Yang et al., 2019), Iodent line PH207 (Hirsch et al., 2016), Lancaster line Mo17 (Sun et al., 2018), W22 (Springer et al., 2018), European Flint lines EP1, F7, DK105, and PE0075 (Haberer et al., 2020), Oh43‐type line PHJ89 (Gage et al., 2019), sweet corn line Ia453‐*sh2* (Hu et al., 2021), and teosinte *Zea mays* ssp. *mexicana* (N. Yang et al., 2017). Structural variation, including copy number variants and their more extreme structural variant, presence–absence variants (PAVs), have been documented in maize and are known to influence phenotypes in a number of crop and model species (Chang et al., 2015; Cook et al., 2012; Gao et al., 2019; Gordon et al., 2017; Hardigan et al., 2016; Hardigan et al., 2017; L. Ou et al., 2018; Pucker et al., 2019; Qi et al., 2013; Song et al., 2020; Wang et al., 2018). Abundant gene content variation exists between the commercial inbreds B73 and PH207 (Hirsch et al., 2016), though syntenic genes are highly conserved between the two lines and differential fractionation plays a limited role in generating gene content variation (Brohammer et al., 2018). Contributions from *Z. mays* ssp. *mexicana* have contributed to modern maize adaptation and improvement (N. Yang et al., 2017), and comparisons between W22 and B73 demonstrated copy number variants of transposable elements (TEs), which influence the study of functional genomics and the impact of TEs on complex phenotypes (Springer et al., 2018). As a result of this pangenome‐level variation, candidate gene predictions can depend on the reference line used for calling single nucleotide polymorphisms (SNPs), and structural variation between reference lines can influence genome‐wide association study results (Gage et al., 2019).

To better understand the genomic diversity present within this important commercial germplasm group and to support ongoing genetic and functional studies, five inbreds that represent the diversity and history of the stiff‐stalk heterotic group (Table 1) were sequenced. All pedigree and accession information was compiled from the Germplasm Resource Information Network Database (npgsweb.ars‐grin.gov). Inbred B84 was released in 1979 as a cycle‐seven selection of the BSSS with *Helminthosporium turcicum* resistance (BSSS(HT)C7, now known as *Setosphaeria turcica*, common name Northern Corn Leaf Blight). Line LH145 is a derivative of B14 through both parents, A632Ht and CM105, and was protected by Holden's Foundation Seed, Inc. in 1984. Line NKH8431, also known as H8431, developed by Northrup, King and Company, was protected in 1988 and was the result of a cross between B73‐like and B14‐like proprietary lines. Line PHB47 was protected via a PVP certificate by PHI in 1984 and was the result of crossing B37 with SD105, an early inbred developed by South Dakota State University. During PHB47's development, populations were backcrossed twice to B37. Finally, PHJ40, the earliest flowering of the group, was developed by PHI by crossing proprietary inbred lines and was protected by PVP certificate in 1987. While the subheterotic groups of the parents of PHJ40 are not known, previous work has shown PHJ40 has admixture‐derived membership with the B37 subgroup (White et al., 2020).

**TABLE 1:** Origins of stiff‐stalk inbred lines described in this study

Line	Originator	Place of origin	Pedigree	PVP^a^ certificate or registration number	Date PVP or registration issued	PI number
B73	Iowa State University	Iowa, United States	Selected from advanced recurrent selection population (C5) of Iowa Stiff Stalk Synthetic (BSSS).	PL‐17	1 Sep. 1972	550473
B84	Iowa State University	Iowa, United States	B84 is a selection from Iowa BSSS(HT)C7 [renamed BS13(S2)C0] that was tested as BS13(S2)CO‐45‐6‐2‐1‐1.	PL‐50	1 July 1979	608767
LH145	Holden's Foundation Seed, Inc.	Iowa, United States	A632Ht × CM105	8300102	29 June 1984	600959
NKH8431 (alias H8431)	Northrup, King & Company	Wisconsin, United States	(377 × B386) × 347. All three parents are Northrup King proprietary lines originating from derivatives of Iowa Stiff Stalk Synthetic. Specifically, 377 derived from Iowa's B73, B386 derived from Minnesota's A632, and 347 derived from Iowa's B14	8800152	30 Nov. 1988	601610
PHB47 (alias B47)	Pioneer Hi‐Bred International, Inc.	Minnesota, United States	B37 × SD105, specifically B37<3‐XX#‐SD105‐#)F21323X11X. B37 is a public inbred line developed from Iowa Stiff Stalk Synthetic at Iowa State University. SD105 is an early public inbred line developed at South Dakota State University.	8300141	26 Oct. 1984	601009
PHJ40	Pioneer Hi‐Bred International, Inc.	Ontario, Canada	B09 × B36, specifically B09/B36)X4122241X	8600133	31 Mar. 1987	601321

*Note*. All information was obtained from the Germplasm Resource Information Network (GRIN).

^a^ PVP, Plant Variety Protection.

**Core Ideas**

- Genome assemblies and annotations of five ex‐PVP stiff‐stalk inbred lines were generated.
- Stiff‐stalk pangenome has limited diversity compared with the overall maize pangenome.
- Stiff‐stalk lines contain distinct haplotypes that have been conserved in breeding germplasm.

## 2 MATERIALS AND METHODS

### 2.1 Genome sequencing and assembly

#### 2.1.1 DNA isolation

High molecular weight DNA was extracted from young leaves using the protocol of Doyle and Doyle (1987) with minor modifications. In brief, young leaves were flash frozen and ground to a fine powder in a frozen mortar with liquid N_2_ followed by very gentle extraction for 1 hr at 50 °C in cetyl trimethylammonium bromide buffer that included proteinase K, polyvinylpyrrolidone‐40, and beta‐mercaptoethanol. After centrifugation, the supernatant was gently extracted twice with 24:1 chloroform/iso‐amyl alcohol. The upper phase was adjusted to one‐tenth volume with 3 M potassium acetate, gently mixed, and the DNA was precipitated with iso‐propanol. DNA was collected by centrifugation, washed with 70% ethanol, air dried for 20 min and dissolved thoroughly in 1 × 10 mM Tris‐Cl, 1 mM ethylene diaminetetraacetic acid at room temperature; DNA size was validated by pulsed‐field gel electrophoresis.

#### 2.1.2 Genome sequencing

Maize inbreds (B84, LH145, NKH8431, PHB47, and PHJ40) were sequenced using a whole genome shotgun sequencing strategy. Sequencing reads were generated using Illumina HiSeq‐2500 and PacBio Sequel I platforms (Supplemental Table S1) at the Department of Energy Joint Genome Institute and the HudsonAlpha Institute. For the PacBio sequencing, an average of 50.8 chips per variety were collected (10‐hr movie time) that yielded 88.4×, 112.2×, 113.7×, 71.2×, and 85.4× coverage for B84, LH145, NKH8431, PHB47, PHJ40, respectively. The Illumina read sets consist of 62.8× to 69.4× coverage of high‐quality Illumina bases for each inbred.

#### 2.1.3 Assembly and integration

The genomes were assembled using the MECAT assembler v1.2 (Xiao et al., 2017) and polished using ARROW v2.2.2 (Chin et al., 2013). To identify false joins, 28,964 nonrepetitive, nonredundant, 1,500‐bp syntenic markers were extracted from the B73 v4 assembly and used to first resolve misjoins and then orient, order, and join the contigs into 10 chromosomes using the B73 markers. Telomeres were evaluated by searching for the kmer (TTTAGGG)_n_, where the value of *n* varied from nine to 20; the longest run of telomere was identified for each contig containing a telomere and placed at the ends of the chromosomes. Remaining scaffolds were screened against the NR GenBank database to remove contamination. Homozygous SNPs and insertion–deletions (INDELs), representing remaining PacBio errors, were corrected using 60× of Illumina reads (2 × 150, 400‐bp insert) by aligning the reads using BWA‐MEM v0.7.15 (Li & Durbin, 2009) and identifying homozygous SNPs and INDELs with the GATK UnifiedGenotyper tool v3.6 (McKenna et al., 2010). The final genome assemblies had 86.6–98.4% of the sequence anchored to the 10 chromosomes with N50 contig lengths ranging from 893.8 kbp to 3.1 Mbp.

### 2.2 Genome quality assessments

#### 2.2.1 Whole‐genome shotgun sequence read alignment

Whole‐genome shotgun (WGS) libraries from the five inbreds were aligned to their cognate genome assemblies (Supplemental Table S2) to assess the quality of the assemblies. Read quality was inspected with FastQC v0.11.8 (http://www.bioinformatics.babraham.ac.uk/projects/fastqc) before processing with Cutadapt v1.18 (Martin, 2011) to remove sequencing adapters and low‐quality reads using the parameters ‘‐q 10 ‐n 2 ‐m 31’. The B73 WGS reads were clipped to 150 nt using the Cutadapt parameters ‘‐u 7 ‐u 93 and ‐U 7 ‐U 93’ prior to adapter trimming. Additionally, processed B73 WGS reads were randomly subsampled with the reformat.sh script from the BBMap suite v37.61 (https://sourceforge.net/projects/bbmap/) using ‘sampleseed = 100’ to obtain similar read quantities as the other libraries. Cutadapt‐filtered WGS reads were aligned to their cognate genome assembly using BWA‐MEM v0.7.16a (Li & Durbin, 2009 ) with the ‘‐M’ flag used to mark shorter split hits as secondary, ‘‐t’ specifying 22 threads, and ‘‐R’ specifying read group headers.

#### 2.2.2 RNA‐sequencing read alignment

Illumina RNA‐sequencing (RNA‐seq) libraries from internode, shoot, leaf, root, and endosperm tissue from each inbred (Li et al., 2020) were used for genome annotation and estimation of expression abundance. Read quality was inspected with FastQC v0.11.8 before processing with Cutadapt v1.18 (Martin, 2011) to remove sequencing adapters and low‐quality reads using the parameters ‘‐q 10 ‐n 2 ‐m 31’. Cutadapt‐filtered RNA‐Seq reads were aligned to their cognate genome assembly using the splice‐site aware algorithm HISAT2 v2.2.0 (Kim et al., 2019) in RF stranded mode with parameters ‘–max‐intronlen 12,000 bp, –dta‐cufflinks, –no‐unal, –no‐summary’.

#### 2.2.3 Benchmarking universal single copy orthologs

Genome assemblies were queried for conserved single‐copy orthologs using benchmarking universal single copy orthologs (BUSCO) (Simao et al., 2015; Waterhouse et al., 2018 ) to assess genic completeness. In addition, genome assemblies of maize lines B73 v4 (downloaded from http://ftp.gramene.org/pub/gramene/release‐59/fasta/zea_mays/), PH207 (downloaded from https://genome.jgi.doe.gov/portal/pages/dynamicOrganismDownload.jsf?organism=ZmaysPH207), Mo17 (downloaded from https://download.maizegdb.org/Zm‐Mo17‐REFERENCE‐CAU‐1.0/), and Ia453‐*sh2* (downloaded from https://www.ncbi.nlm.nih.gov/assembly/GCA_016432965.1) were also queried. BUSCO v4.1.4 was run in genome mode using the Embryophyta odb10 dataset (creation date, 2019‐11‐20; number of species, 50; number of BUSCOs, 1614) with default parameters.

#### 2.2.4 Long‐terminal repeat assembly index

The assembly contiguity of the TE space of each genome was evaluated using long‐terminal repeat (LTR) assembly index (LAI) (beta 3.2) (S. Ou et al., 2018) from the LTR_retriever v2.9.0 package (Ou & Jiang, 2018) with parameters ‘‐intact file4 ‐all file3 ‐q ‐totLTR 76.34 ‐iden 94.854 ‐t 10’. The ‘‐intact’ file was generated using Extensive de‐novo TE Annotator (EDTA) v1.9.0 (Ou et al., 2019) as described below. The ‘‐all’ file was the RepeatMasker out file of each genome annotated by the pan‐stiff‐stalk TE library (see section 2.3.2 for details on generation of the library).

### 2.3 Genome Annotation

#### 2.3.1 Construction of the pan‐stiff‐stalk TE library

A manually curated TE library from the Maize TE Consortium (MTEC, downloaded from https://github.com/oushujun/MTEC) (Schnable et al., 2009) was used as the base library and supplemented with novel TE families identified from the six genomes including the five stiff‐stalk genomes reported in this study and the B73 v4 genome. The EDTA package v1.9.0 (Ou et al., 2019) was used to identify novel TEs of each genome with parameters ‘–cds’ and ‘–curatedlib’. With the ‘–cds’ parameter, coding sequences annotated from each genome were provided to remove gene‐related sequences in the resulting TE library. With the ‘–curatedlib’ parameter, the base library (i.e., the MTEC library) was provided for EDTA to identify novel TE families beyond those already present in the MTEC library. The six novel TE libraries were combined with the MTEC library using the Perl script ‘make_panTElib.pl’ in the EDTA package. The 80‐95‐80 rule (80% identity, 95% coverage, 80 bp minimum length) was used to cluster redundant sequences with parameters ‘‐miniden 80 ‐mincov 0.95 ‐minlen 80’.

#### 2.3.2 Annotation of pangenome TEs

Transposable element annotation of each genome was performed based on both structural and homology annotations using EDTA v1.9.0 (Ou et al., 2019) and RepeatMasker v4.0.9 (http://www.repeatmasker.org/). First, each genome was annotated using the pangenome TE library and RepeatMasker with parameters ‘‐q ‐no_is ‐norna ‐nolow ‐div 40’, allowing up to 40% sequence divergence. The EDTA was executed again on the original structural annotation of each genome to unify TE family names with parameters ‘–cds file1 –curatedlib file2 –step anno –rmout file3 —anno 1 –evaluate 1’. The ‘–cds’ file was the same coding sequences for each genome previously provided. The ‘–curatedlib’ file was the pan‐stiff‐stalk TE library. The ‘–rmout’ file was the RepeatMasker out file of each genome annotated by the pan‐stiff‐stalk TE library. The insertion time of each LTR retrotransposon was estimated by LTR_retriever v2.9.0 (S. Ou et al., 2018) with *T* = *K*/2μ, where *K* is the divergence between the left and right LTR of the element and μ = 3.3 × 10^−8^ bp^−1^ yr^−1^ for heterochromatic regions (Clark et al., 2005).

#### 2.3.3 Annotation of gene models

Each of the six stiff‐stalk genomes, including B73, were annotated for gene models using an identical pipeline using inbred‐specific transcript evidence, thereby eliminating false annotations from transcripts from other inbreds. The RNA‐seq libraries were cleaned using Cutadapt v2.9 (Martin, 2011) using the parameters ‘–times 2 –minimum‐length 100 –quality‐cutoff 10’. Cleaned reads from each library were then aligned to their respective genome assembly using HISAT2 v2.2.0 (Kim et al., 2019) with the parameters ‘–max‐intronlen 5000 –rna‐strandness RF –no‐unal –dta’, and assembled using Stringtie v2.1.1 (Kovaka et al., 2019) with the parameter ‘–rf’ and the assembled transcript sequences extracted with gffread v0.11.7 (Pertea & Pertea, 2020).

Each genome assembly was masked with RepeatMasker v4.1.0 (http://www.repeatmasker.org/) using the curated maize repeat library maizeTE02052020 (https://github.com/oushujun/MTEC) using the parameters ‘‐e ncbi ‐s ‐nolow ‐no_is ‐gff’. Augustus v3.3.3 (Stanke et al., 2008) was used to generate gene predictions on the masked assemblies using the maize5 training parameter set and the RNA‐Seq alignments as hints. The gene predictions were refined using PASA2 v2.4.1 (Haas et al., 2005) (http://pasapipeline.github.io/) in two rounds of annotation comparison (‐I 60000) using the RNA‐Seq transcript assemblies as evidence to generate the working model gene set.

To identify high‐confidence gene models, the working gene model set was searched against the PFAM database v32 (Finn et al., 2016) with hmmscan (HMMER, v3.2.1) (Mistry et al., 2013) with a cutoff of ‘–domE 1e‐3 ‐E 1e‐5’ to identify gene models encoding a Pfam domain as described previously (Pham et al., 2020). Gene expression abundances for the working gene models (transcripts per million) were generated for each RNA‐seq library using Kallisto v0.46.0 (Bray et al., 2016). High confidence gene models were identified if they had a transcripts per million value > 0 in at least one RNA‐seq library and had a PFAM domain match. Partial‐gene models or gene models with matches to TE‐related PFAM domains were also excluded from the high‐confidence model set.

Functional annotation was assigned to the working gene model set using search results from the predicted proteins against the *Arabidopsis* proteome (TAIR10; Arabidopsis.org), the PFAM database v32 (Finn et al., 2016), and Swiss‐Prot plant proteins (release 2015_08). Results were processed in the same order (TAIR, PFAM, Swiss‐Prot) and the function of the first informative hit was transitively assigned to the gene model.

### 2.4 Comparative genome analyses

#### 2.4.1 Transcript alignment

Annotated high‐confidence coding sequences (CDS) from all six genomes (B73, B84, LH145, NKH8431, PHB47, and PHJ40) were aligned to all six genome assemblies using GMAP v20170905 (Wu & Watanabe, 2005) with thresholds of 95% identity and 95% coverage used to determine gene presence or absence. Sequences were considered present in a genome assembly if they aligned to either a unique location or multiple locations.

#### 2.4.2 Structural variation

Structural variants (SVs) for the stiff‐stalk genomes were characterized as described previously (Hufford et al., 2021). Briefly, this SV‐detection pipeline includes a combination of three different methods using three different data types mapped against the B73 v4 reference (Jiao et al., 2017). The first approach involved mapping long reads from each genome to B73 v4; the second, aligning the chromosomal genome assemblies of each line to B73 v4; and the third, taking in silico digested assemblies (to simulate a BioNano optical map) of each maize line and aligning these to the simulated B73 optical map. These approaches were used to characterize SVs separately and then collapsed to generate a comprehensive set of SVs for the five stiff‐stalk inbreds.

Error‐corrected long reads of each stiff‐stalk maize inbred were mapped to the B73 v4 genome using a sensitive mapping program, NGMLR v0.2.7 (Sedlazeck et al., 2018). All options were set to default, except for the ‘–presets’ option, which was set to ‐pacbio’, and the ‘–bam‐fix’ option, which enables bam compatible output files. In order to accelerate the mapping step, input files (PacBio reads) were split into smaller subsets, and mapping was performed in parallel to the reference genome followed by concatenation of bam files to a single file using SAMtools merge v1.9 (Li et al., 2009). The final BAM file for each maize line was then used with SNIFFLES v1.0.11 (Sedlazeck et al., 2018) to call SVs in two iterations. For the first iteration, SNIFFLES was run using stringent parameters ‘–max_num_splits 2, –min_support 20, –min_zmw 2, –min_seq_size 5000, –max_distance 5000, –cluster, and –cluster_support 2’ with minimum SV size set to 100 bp ‘–min_length 100’ and a VCF‐formatted file generated for each maize inbred line. SURVIVOR v1.0.6 (Jeffares et al., 2017) was then used to merge individual VCF files, with options max distance between breakpoints set to 1000 and taking SV type and strand into account. We did not use the options to estimate SV size nor to take the minimum size of SV into account in order to generate a joint SV VCF file. Missing and absent SV calls across lines were filled in a second iteration of SNIFFLES. For this run, the merged SVs were provided as input (–Ivcf) along with the original BAM files (mapped reads). The final genotyped SVs were then once again combined using SURVIVOR and filters were applied to limit SVs to a size range of 100 bp to 100 kbp.

Each of the stiff‐stalk inbred assemblies was aligned against the B73 v4 reference, using minimap2 v2.17‐r941 (Li, 2018) to generate PAF‐formatted alignment files (default options with ‘‐c’ to enable cigar strings in the output files, ‘‐x asm5’ to use a ∼0.1% sequence divergence preset, and ‘–cs’ to encode bases at mismatches and the INDELs options). The PAF files were sorted using the UNIX sort command, and INDELS were inferred using paftools (k8 paftools.js call) (Li, 2018). The native, tab‐separated output files were converted to BED format using awk in order to visualize INDELS and syntenic blocks in the IGV genome browser (Robinson et al., 2011).

For larger SVs (>100 kbp) that could not be characterized using long reads or aligned using genome‐to‐genome‐based alignment methods, we used optical‐map‐based SV detection. In this approach, the maize genome was first subjected to in silico digestion using the fa2cmap_multi_color.pl script in the BioNano solve program and the CTTAAG enzyme motif in order to simulate a contiguous Bionano optical map for each chromosome. Second, CMAP format BioNano maps were aligned against the B73 CMAP file using the RefAligner tool from runCharacterize.py and the runSV.py script from BioNano solve. Since labeled markers are aligned instead of individual bases, accurate detection of large‐scale inversions, deletions, and insertions can be achieved; however, smaller SVs are difficult to detect. Default options were used for both steps, with the arguments supplied through an XML file (optArguments_nonhaplotype_noES_DLE1_saphyr.xml). In the third step, the resulting smap file from the second step (with the list of structural variants detected between query maps and reference maps in tsv format) was converted to VCF formatted files using the smap_to_vcf_v2.py script. The final SV file in VCF format was filtered to only include SVs >100 kbp using an awk command. BioNano‐based SV identification was carried out using two different enzymes and the breakpoints were manually inspected using bed files generated from genome‐to‐genome alignments in IGV and synteny dot plots before finalizing the SV calls. The BioNano SV start and stop sites were refined based on the consensus positions determined by two enzymes independently along with genome‐to genome alignments. The final curated SVs were merged to generate a joint SV file using SURVIVOR, with similar options as detailed above. The final SV set was generated by merging the SNIFFLES SVs with the curated BioNano SVs.

To characterize SVs within the 9–11 kbp size class, which was enriched in deletions, we annotated these sequences with the pangenome TE library using RepeatMasker and assessed enrichment for full‐length LTR (fl‐LTR) retrotransposons, which typically fall in this size range, using the 80‐80‐80 rule that required at least 80 bp, 80% identity, and 80% coverage for the matching LTR sequence. To assess the expectation of fl‐LTRs found in 9–11 kb random genomic sequences, we extracted random sequences mimicking the exact length of these deletions in the B73v4 genome; this random process was iterated 10 times.

#### 2.4.3 Syntenic analysis of gene content across the inbreds

Syntenic regions among the six stiff‐stalk inbreds were identified using the MCScanX v20170322 toolkit (Wang et al., 2012). The MCScanX algorithm was run with default parameters on each inbred using B73 v4 MSU annotation generated in this study as the reference to determine collinear blocks of genes.

#### 2.4.4 Orthology and paralogy analysis

Orthologous and paralogous genes among the six stiff‐stalk genomes were identified using Orthofinder v2.5.1 (Emms & Kelly, 2019). Analyses were conducted using the predicted proteomes from each stiff‐stalk genome with default settings. Orthologous groups represented by all accessions were used to construct and root a consensus tree with the STAG and STRIDE algorithms, respectively (Emms & Kelly, 2017, 2018).

### 2.5 Resistance gene classification

Putative resistance genes were identified by querying high‐confidence representative peptides against the curated Pathogen Receptor Genes database (PRGdb) v3.0 using the DRAGO2 API (Osuna‐Cruz et al., 2018).

### 2.6 Identification of descendant regions

Single nucleotide polymorphisms generated using RNA‐seq data with imputation of the 942 accessions in the Wisconsin Diversity panel (WiDiv‐942) (Mazaheri et al., 2019) were used to generate haplotypes using the TASSEL 5.0 plugin FILLINFindHaplotypesPlugin (Bradbury et al., 2007). Default parameters were used except for the parameters ‘‐mxDiv 0.03, ‐minTaxa 1, ‐hapSize 1000, ‐minPres 250, and ‐extOut true’. Thus, maximum divergence from the founder was set to 3%, the minimum number of taxa set to one to allow haplotypes found in a single individual, and the haplotype size was set to 960 SNP windows, as 960 is the closest multiple of 64 less than 1000. Haplotype data was processed and assigned to a hierarchy using the ‘convert_fillinhaps_to_feather_or_csv.R’ and ‘apply_hierarchy.R’ scripts (Coffman et al., 2020). This script names a representative inbred for each haplotype group based on a hierarchy such that the highest ranking inbred within each group is listed as the representative. Ranking inbreds using a hierarchy allows for more convenient visualization of shared haplotype blocks and transmission through time and selection.

The WiDiv panel contains 15 inbreds that represent 13 of the original 16 BSSS founders in addition to the parents of one of the unavailable lines. Inbreds Ind‐461‐3 and CI617 were not available, while inbreds B2 and Fe were included as the parents of unavailable inbred F1B1 (Gerke et al., 2015). A group of 41 unselected inbreds from the base BSSS population, hereafter BSSSC0, followed in the hierarchy. Previous work identified within the WiDiv 16 public inbreds that were classified according to pedigree information as directly selected from the BSSS germplasm and 21 ex‐PVP inbreds that were derived from the stiff‐stalk founders B14, B37 and B73 according to ADMIXTURE analysis (Gage et al., 2019). These lines followed the BSSSC0 inbreds in our hierarchy, followed by any other remaining inbreds that clustered with stiff‐stalk founders B14, B37, and B73 according to ADMIXTURE analysis (Mazaheri et al., 2019). Lines were placed in order of year of release when that information was available, otherwise, lines were placed in the hierarchy in alphanumeric order within their groups. In addition, any haplotype groups that were represented by nonfounder or BSSSC0 lines were set to be plotted in white so that only haplotypes that were present in the base BSSS population would be plotted in color in the publicly released and ex‐PVP lines. Once the hierarchy was constructed, a neighbor‐joining tree was made using default parameters in TASSEL 5.0 to order the inbreds along the *x* axis according to genetic distance to facilitate visualization of shared haplotypes (Bradbury et al., 2007).

Allele frequencies were calculated for the base, unselected population, consisting of the founder and BSSSC0 lines, and for the selected population, consisting of the public and ex‐PVP lines identified previously (Gage et al., 2019). The *F*_ST_ value was calculated using vectorFst.R (Beissinger et al., 2014) available at http://beissingerlab.github.io/docs/vectorFst.R, including a correction for the small number of populations (Weir et al., 1984). The SNPs were binned into the same windows used for haplotype analysis, and the window took on the maximum *F*_ST_ value of SNPs within the window. Windows in the top 10th percentile of genome‐wide values were plotted in black alongside haplotypes for visualization.

Finally, SNP‐based identity by state (IBS) was calculated using the WiDiv‐942 RNA‐seq SNPs for the five inbreds compared with their most‐related founder stiff‐stalk lines. Values were averaged into bins using the same physical position boundaries as the previous haplotype plots. Approximate centromere locations, as determined by the mean physical position of the centromere in the maize B73‐Ab10 assembly, are marked by vertical lines on each chromosome (Liu et al., 2020). Windows were noted as conserved if the average IBS was >0.97.

## 3 RESULTS & DISCUSSION

### 3.1 Assembly of five stiff‐stalk genomes

High‐quality assemblies were generated for five stiff‐stalk founder lines—B84, LH145, NKH8431, PHB47, and PHJ40—from approximately 124.2 million PacBio reads (Table 2). Assembly sizes ranging from 2.13 (NKH8431) to 2.18 Gbp (LH145) are comparable to previous PacBio assembly sizes of 2.13, 2.2, and 2.29 Gbp for B73 v4 (Jiao et al., 2017), Mo17 (Sun et al., 2018), and la453‐*sh2* (Hu et al., 2021), respectively. Each stiff‐stalk assembly had N50 contig lengths ranging from 894 kbp (PHJ40) to 3.1 Mbp (B84), with the largest contig measuring 18.4 Mbp and N50 scaffold lengths exceeding 200 Mbp. On average, 94.5% of the assemblies were anchored to the 10 maize chromosomes.

**TABLE 2:** Genome assembly metrics for six stiff‐stalk inbreds

Parameter	B73 v4.0^a^	B84 v1.0	LH145 v1.0	NKH8431 v1.0	PHB47 v1.0	PHJ40 v1.0
PacBio coverage	65×	88.4×	112.3×	113.7×	71.2×	85.4×
PacBio average read length (kbp)	11.7	8.4	6.1	6.2	6.9	6.1
PacBio reads (millions)	34.7	19.3	28.8	33.8	17.1	25.3
Scaffolds^b^						
Genic content (%)	46.9	46.9	46.9	46.9	46.9	46.9
Total number of scaffolds	265	291	1584	380	930	1547
Scaffold sequence (Mbp)	2,134.4	2,131.4	2,181.9	2,125.1	2,155.6	2,153.8
Scaffold N50 size (Mbp)	223.9	218.2	219.3	212.8	212.8	202.6
Scaffold L50 number	5	5	5	5	5	5
Breaks^c^	–	92	67	167	117	107
Joins^c^	–	1,184	2,115	1,626	2,908	2,702
Largest scaffold length (Mbp)	307	305	309	310	296	271
Contigs^b^						
Total no. of contigs	2,785	1,475	3,699	2,006	3,841	4,250
Contig sequence (Mbp)	2,103.6	2,119.5	2,160.7	2,108.9	2,126.5	2,126.8
Contig N50 size (Mbp)	1.3	3.1	1.5	2.0	1.0	0.9
Contig L50 number	505	182	388	280	568	682
Largest contig length (Mbp)	7.3	18.4	10.0	15.6	8.5	7.8
Proportion of assembly on chromosomes^b^ (%)	98.7	98.4	95.0	97.7	95.1	86.6

^a^ B73 v4 assembly was sourced from Gramene release 59.

^b^ Metrics do not include plastid sequences.

^c^ Misjoins identified by an abrupt change in B73 linkage group were corrected by creating breaks in the assembly.

A high proportion of WGS reads aligned to their cognate assembly; >99.8% of WGS reads aligned to the non‐B73 stiff‐stalk genome assemblies, and 96.1% of B73 WGS reads aligned to the B73 v4 genome assembly (Supplemental Table S2). Properly paired reads accounted for 94.3 (B73) to 98.7% (PHJ40) of the total alignments. The proportion of reads mapping to multiple genomic locations ranged from 12.0% in the B73 library to 15.7% in the B84 libraries.

With respect to genic content, a high proportion of RNA‐seq reads aligned to their cognate assembly regardless of inbred or tissue (Supplemental Table S3). The average alignment rate of the RNA‐seq reads from the five tissues to their cognate genome assemblies was >93.0% for all inbreds. The B84 root tissue was the only library with low alignment rate (80.0% of reads aligned). A megaBLASTn query of the B84 root tissue alignment file against the NCBI nt nucleotide sequence database (http://ftp.ncbi.nlm.nih.gov/blast/db/; accessed 11 Feb. 2019) with an *e*‐value threshold of 1×10^−20^, 90% identity, and 50% coverage did not detect widespread contamination. Further investigation of the B84 root tissue alignment file revealed a large spike of deletions on the reverse read occurring in the 33rd sequencing cycle that may have negatively impacted alignment. All six stiff‐stalk genome assemblies (B73 v4, B84, LH145, NKH8431, PHB47, and PHJ40) showed a high proportion of complete BUSCO orthologs, with very few orthologs categorized as fragmented or missing (Supplemental Table S4). The stiff‐stalk assemblies contained comparable amounts of single‐copy BUSCO orthologs, ranging from 1,548 (95.9%) in B84 to 1,558 (96.6%) in PHJ40. These metrics are comparable to other PacBio‐derived maize assemblies for B73 v4 (Jiao et al., 2017), Mo17 (Sun et al., 2018), and Ia453‐*sh2* (Hu et al., 2021), containing 1,551; 1553; and 1562 single‐copy orthologs, respectively. Furthermore, <3% of the ortholog set was classified as fragmented or missing in any stiff‐stalk genome assembly, reflecting a high coverage of genic space.

Transposable elements are one of the most difficult components to assemble in plant genomes because of their repetitiveness and low divergence (Ou et al., 2019). We evaluated the contiguity of the TE space using the LAI software (S. Ou et al., 2018). Relatively high LAI values were observed across the assemblies, with an average of 26.78 (Supplemental Table S5), which falls into the “gold” quality category, as previously defined (S. Ou et al., 2018). Regional LAI values of the pseudomolecules were consistently high across each chromosome (Supplemental Figure S1). The LAI of the assembled chromosomes was, on average, 79 times higher than those of the unplaced scaffolds (Supplemental Figure S2), indicating substantially decreased contiguity of the TE space in the unplaced scaffolds relative to those that were placed into chromosomes.

### 3.2 Transposable element composition

Transposable elements were annotated first based on structural features and then based on homology to a pan‐stiff‐stalk TE library. The pan‐stiff‐stalk TE library was constructed using the manually curated library from the MTEC (Schnable et al., 2009) as the base with the addition of novel TE sequences from each stiff‐stalk genome. In each of the assemblies, ∼87% of the genome was annotated as TEs (Supplemental Table S6). The LTR retrotransposons contributed the most (average of 75.69%), with *Gypsy* and *Copia* elements contributing 46.69 and 25.26% to the genome size, respectively (Supplemental Table S6). Approximately 50,000 intact LTR retrotransposons were identified in each genome, and more than half of these (55.5%) were younger than 150,000 yr old (Supplemental Figure S3), suggesting active amplification of LTR retrotransposons and a relatively short life cycle of these elements. DNA TEs contributed 11.12% to genome size, on average, with *CACTA* and *Helitrons* representing the most sizable DNA TE superfamilies at 3.64 and 3.51% of genomic content, respectively (Supplemental Table S6). Non‐TE interspersed repeats (i.e., centromere, subtelomere, rDNA, and knobs) contributed to only 0.23% of the assemblies, which is probably an underestimate in light of challenges in assembling these repetitive sequences (Ou et al., 2020).

### 3.3 Annotation of six stiff‐stalk genomes

The six stiff‐stalk genomes were annotated in parallel using ab initio gene predictions in combination with empirical, inbred‐specific transcript evidence from a core set of diverse tissues (leaf, internode, root, shoot, and self‐pollinated endosperm) (Table 3). This approach ensured that the resulting gene annotation for each stiff‐stalk inbred was not confounded by gene models and transcript evidence from other accessions, which have been shown to differ significantly in maize (Hirsch et al., 2016) and *Arabidopsis thaliana* (L.) Heynh. (Gan et al., 2011). In addition to the five ex‐PVP inbreds described above, we also annotated the B73 v4 reference genome assembly (hereafter referred to as B73 v4 MSU). The current annotation of the B73 v4 assembly (Jiao et al., 2017) incorporates an enormous set of publicly available transcript sequences generated across multiple platforms from multiple inbreds using a MAKER‐P pipeline that resulted in a significant overannotation of gene model isoforms. For example, there are 161,680 working transcripts in the B73 v4 Gramene annotation (Jiao et al., 2017), yet 73,362 transcripts in the B73 v4 MSU annotation, a number comparable to the 72,635–75,124 transcripts present in the B84, LH145, NKH8431, PHB47, and PHJ40 genomes (Table 3). Therefore, any direct comparison of the B73 v4 Gramene annotation to the five stiff‐stalk genome described in this study would be confounded because of the nearly double the number of transcripts present in the B73 v4 Gramene annotation. Thus, through the use of a core set of representative tissues specific to each of the six stiff‐stalk genomes and a streamlined annotation pipeline, we have minimized the frequency of unsupported isoforms. Furthermore, this permits direct comparisons between all of the six stiff‐stalk genomes and a reduction of artifacts associated with differential annotation methods.

**TABLE 3:** Gene annotation metrics of six stiff‐stalk inbred genomes

Parameter	Stiff‐stalk genome						
	B73 v4	B73 v4	B84^a^	LH145^a^	NKH8431^a^	PHB47^a^	PHJ40^a^
Annotation	Jiao et al., 2017	MSU^c^	MSU^c^	MSU^c^	MSU^c^	MSU^c^	MSU^c^
Working set							
Number of genes	49,085	49,986	50,861	52,133	50,732	50,982	51,335
Number of gene models	161,680	73,362	74,587	75,124	73,946	72,635	74,593
Mean gene model length (bp)	–	1,583	1,557	1,554	1,563	1,556	1,592
Median gene model length (bp)	–	1,350	1,321	1,319	1,327	1,309	1,335
High confidence set							
Number of genes	39,324	39,252	40,253	40,968	40,478	40,040	40,431
Number of gene models	131,319	62,091	63,430	63,451	63,114	61,156	63,110
Mean gene model length (bp)	–	1,766	1,730	1,736	1,734	1,742	1,777
Median gene model length (bp)	–	1,549	1,509	1,517	1,519	1,514	1,541
Representative/primary gene model							
Number of gene models^b^	39,498	39,252	40,253	40,968	40,478	40,040	40,431
Mean gene model length (bp)	1,584	1,476	1,455	1,457	1,452	1,464	1,470
Median gene model length (bp)	1,323	1,267	1,233	1,243	1,237	1,251	1,248

^a^ Assembly provided in this paper.

^b^ B73 metrics include 174 plastid gene models.

^c^ MSU, Michigan State University. Annotation provided in this paper.

### 3.4 Genome variation of six stiff‐stalk genomes

**FIGURE 1:**
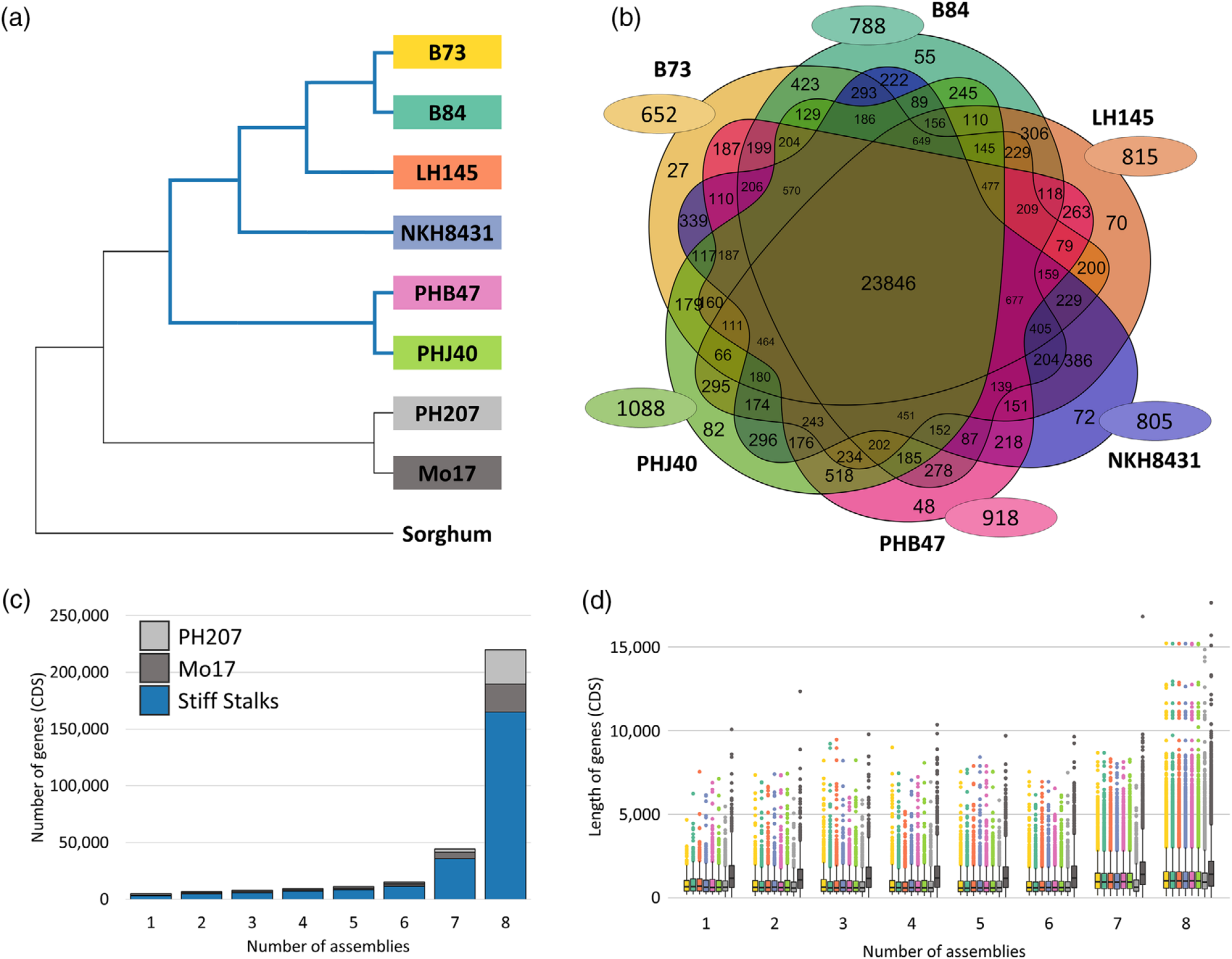
Stiff‐stalk panproteome and pantranscriptome. Predicted proteomes for six stiff‐stalk inbreds, Mo17 and PH207, and *Sorghum bicolor* were assigned orthologous groups using Orthofinder v2.5.1 (Emms & Kelly, 2019). (a) Cladogram showing the relationships among proteomes. The cladogram was constructed and rooted from ancestral orthologous groups with the STAG and STRIDE algorithms (Emms & Kelly, 2017, 2018 ), respectively. Inbred lines belonging to the stiff‐stalk lineage are indicated in blue. All branches had multiple sequence alignment support values of 100%. (b) Venn diagram of orthologous and paralogous group occupancy across six stiff‐stalk inbreds. Intersections indicate orthologous groups containing at least one gene from a given stiff‐stalk inbred. There were 23,846 ‘core’ orthologous groups containing at least one protein from all stiff‐stalk inbreds, representing 55.57% of the total orthologous groups assigned (including singletons). Similarly, 31.83% of orthologous groups were missing at least one stiff‐stalk inbred, and 12.62% of paralogous groups were unique to a stiff‐stalk inbred (i.e., inbred‐specific paralogs plus singletons). The number of singletons for each stiff‐stalk inbred is shown in an ellipse overlaying the Venn diagram. (c) High‐confidence, representative coding sequences (CDS) of six stiff‐stalk inbreds, PH207 and Mo17, were aligned to the eight genome assemblies to assess presence–absence based on DNA sequence alignments with GMAP (Wu & Watanabe, 2005). (d) The length distribution of CDS considered present in one through eight assemblies are shown. Boxplots are colored according to inbred line as depicted in (a)

Variation in the six stiff‐stalk assemblies was examined at the gene and genome level. First, the relationship between six stiff‐stalk inbreds, two inbreds outside the stiff‐stalk heterotic pool (Mo17 and PH207), and sorghum [*Sorghum bicolor* (L.) Moench] was determined using a cladogram generated from orthologous groupings (Figure 1a). All branches had multiple sequence alignment support values of 100%. As expected, sorghum was distantly related to the maize lines, and Mo17 and PH207 inbreds clustered separately from the six stiff‐stalk inbreds. Among the stiff‐stalk inbreds, B73 and B84 were closely related, while the PHI inbreds PHB47 and PHJ40 clustered together separately from the other inbreds.

To better understand the stiff‐stalk panproteome, we examined the presence of orthologous and paralogous groups within the predicted proteomes of the six stiff‐stalk lines (Figure 1b). A total of 236,356 genes (97.90% of all input genes) were assigned to 37,866 orthologous and paralogous groups, while the remaining predicted proteins were considered singletons. Very few stiff‐stalk proteins were assigned to paralogous groups (0.47–1.01%) or classified as singletons (1.66–2.69%), further reflecting the similarities between their predicted proteomes. The 23,846 ‘core’ orthologous groups containing at least one gene from all of the stiff‐stalk lines made up 55.54% of the orthologous and paralogous groups and singletons, while 31.83% of orthologous groups were missing orthologs from one or more stiff‐stalk lines and were considered ‘shell’ orthologous groups (Supplemental Table S7). The ‘cloud’ groups were composed of inbred‐specific paralogous groups (*n* = 354) and singletons (*n* = 5,066) across all six inbreds (Supplemental Table S7). In terms of proteins, the ‘core’, ‘shell,’ and ‘cloud’ orthologous groups contained 74.65, 22.59, and 2.76% of the total predicted proteins, respectively. Inbred line PHJ40 contained the most inbred‐specific paralogous groups and proteins (*n* = 1,170 groups, 1,496 proteins), while B73 contained the fewest (*n* = 697 groups, 836 proteins).

Next, to look at the nucleotide sequence conservation among the stiff‐stalk genomes directly rather than protein level conservation, we aligned the CDS of the high‐confidence representative gene models from each stiff‐stalk inbred to each stiff‐stalk genome assembly. Genes were considered present in an inbred if they aligned to a unique location or multiple locations in the target genome. As expected, cognate gene alignments showed the highest proportion of genes classified as present (average of 99.66%). Among the six stiff‐stalk inbreds, the lowest proportion of genes present occurred when aligning PHJ40 genes to B84 (89.21%) and the highest proportion of genes present occurred when aligning B73 v4 MSU genes to B84 (Supplemental Table S8). The PHJ40 and PHB47 gene sets contained slightly lower proportions of ‘present’ genes (89.58–90.13%) when aligned to the other stiff‐stalk assemblies. Considering that the annotated genes in each of the six stiff‐stalk lines contained similar proportions of BUSCO‐derived orthologs (Supplemental Table S4), the relatively low alignment of PHJ40 and PHB47 could reflect subtle divergence from the other stiff‐stalk lines. The Orthofinder cladogram supports this hypothesis, as PHB47 and PHJ40 were not in the same clade as B73, B84, LH145, and NKH8431 (Figure 1a). With respect to the stiff‐stalk pangenome, of the 241,034 stiff‐stalk pangenes that were present in at least one assembly, 80.38% were considered ‘core’ genes present in all six stiff‐stalk lines, and the ‘shell’ and ‘cloud’ proportions were 17.79 and 1.83%, respectively (Supplemental Table S9). The proportions of pangene designations are comparable to those from the stiff‐stalk panproteome analysis, yet a greater proportion of genes were classified as ‘core’ using the representative gene model CDS alignments compared with the orthologous pangenes (80.38 and 74.65%, respectively) because of the inherent differences in nucleotide‐ and protein‐level variation. The stiff‐stalk pangenome analyses had substantially fewer cloud genes than reported previously in analyses of the pangenome of larger diversity panels (Hirsch et al., 2014; Gage et al., 2019) or in comparison of B73 to PH207 (Hirsch et al., 2016) consistent with the higher degree of diversity and divergence between those inbreds, respectively, relative to this panel composed solely of stiff‐stalk lines.

To better understand the relationship between the stiff‐stalk genome and other heterotic pool pangenomes, we examined two additional inbred lines, PH207 and Mo17, which represent the Iodent and Lancaster heterotic pools, respectively. As the methods used to annotate Mo17, PH207 and the six stiff‐stalk inbreds differed, we limited our analyses of the pangenome in the stiff‐stalk, Iodent, and non‐stiff‐stalk heterotic pools to alignments of representative gene model coding sequences to the eight genome assemblies. At the gene level, the stiff‐stalk genes were less likely to be present in the PH207 and Mo17 assemblies and vice versa (Supplemental Table 8). Notably, only 78.70% of the stiff‐stalk genes were present in the PH207 assembly compared with 87.03% of PH207 genes found among the stiff‐stalk assemblies, which may indicate true divergence of PH207 but also the incompleteness of the PH207 assembly which was generated from short reads (Hirsch et al., 2016). In comparison, 88.73% of stiff‐stalk genes aligned to the Mo17 assembly, with PHJ40 genes in particular aligning slightly more often to Mo17 (90.33%) than to the other stiff‐stalk lines (89.58%). Even so, 68.77% of genes were present in all eight inbred assemblies and considered ‘core’, 13.84% were present in seven assemblies, and 4.73% were present in at least six assemblies; in total, 29.69% of the genes were present in two to seven assemblies representing ‘shell’ genes (Figure 1c; Supplemental Table S10). Overall, 98.46% of the genes were either ‘core’ or ‘shell’ in comparison to just 1.54% of the total genes that aligned only to a single assembly (‘cloud’). Core genes, present in all eight assemblies, as well as shell genes present in seven assemblies, were longer on average than genes found in six or fewer assemblies (Figure 1d), consistent with previous observations about gene length and membership in the pangenome (Gordon et al., 2017). Differences in gene complement between heterotic pools have been hypothesized to contribute to the heterosis observed in hybrids yet incompleteness in the genome assemblies, especially in the case of PH207, and differences in gene annotation methods can impact precise detection of allelic variants resulting in overestimations of the dispensable portion of the pangenome. Future studies with a broader set of inbred lines from the non‐stiff‐stalk and Iodent heterotic pools will permit assessment of the extent of inbred‐ and heterotic‐pool‐specific genes.

**FIGURE 2:**
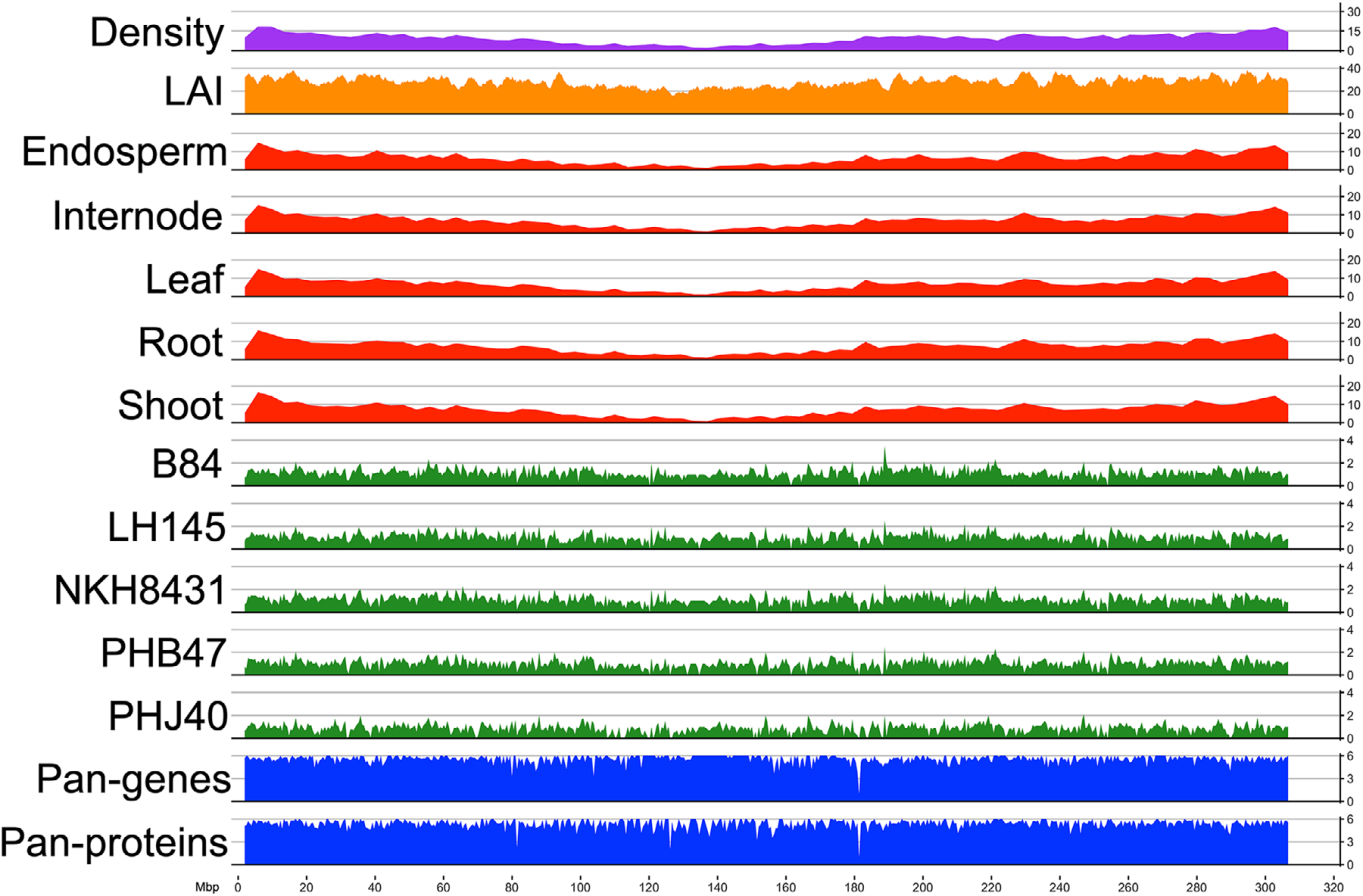
Gene density, gene expression, and syntelogs on *Zea mays* B73 chromosome 1. The number of high‐confidence representative gene models is displayed in purple, long‐terminal repeat assembly index (LAI) values are displayed in orange, and the number of expressed high‐confidence, representative gene models across five tissues is displayed in red. The presence–absence of syntelogs among the other stiff‐stalk inbreds is displayed in green, and the average number of pangenes and panproteins across the non‐B73 stiff‐stalk inbreds are displayed in blue. Genic data were binned into 500‐kbp nonoverlapping windows for visualization

For synteny analysis, B73 was selected as the reference stiff‐stalk genome to which the other stiff‐stalk assemblies were compared. As expected, B73 gene density was elevated on the arms of the chromosome with gene expression mirroring gene density (Figure 2). Collinear blocks were identified for each stiff‐stalk inbred compared with B73, revealing high levels of collinearity (Figure 2; Supplemental Table S11). In total, 1,178 (B84) to 1,737 (PHJ40) collinear blocks were detected across the five stiff‐stalk inbreds, containing 45,741 (PHJ40) to 53,895 (B84) syntenic gene pairs, or syntelogs. (Supplemental Table S11). The detection of ∼500 more collinear blocks and 8,000 fewer syntelogs in inbred line PHJ40 is attributable to its distance from B73 and its more fragmented genome assembly. The collinear blocks composed of chromosome–chromosome alignments made up 61.95 (PHJ40) to 65.03% (B84) of the total collinear blocks in each stiff‐stalk line and contained 77.05 (PHJ40) to 84.75% (B84) of all syntelogs, demonstrating the genic content of the stiff‐stalk lines is present on the assembled pseudomolecules rather than unplaced contigs. The mean and maximum number of genes in these collinear blocks was largely consistent, with four of the five assemblies averaging 56 syntelogs per block and a maximum block size of 4,376 syntelogs compared with PHJ40 with an average of 33 syntelogs per block and a maximum block size of 1,120 syntelogs. The number of syntenic genes across B73 and each comparator stiff‐stalk inbred detected by the synteny analysis ranged from 55,427 genes in the B73‐PHJ40 comparison to 62,951 genes in B84‐PHJ40 comparison. These genes made up 92.66–99.35% of all syntenic gene pairs found among chromosome–chromosome collinear blocks, which further reflects the high conservation of genic content among the stiff‐stalk inbred lines.

**FIGURE 3:**
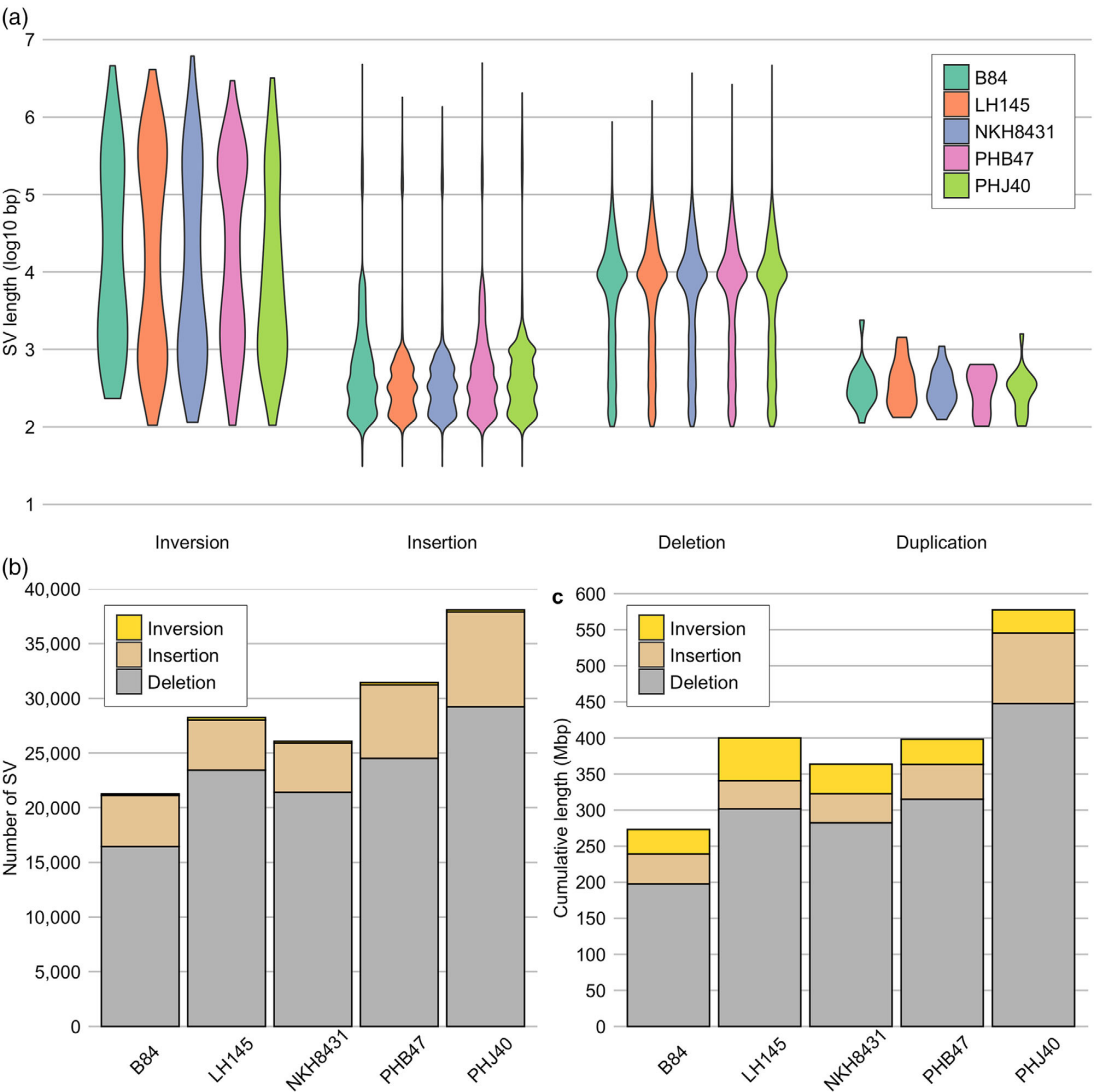
Structural variants (SVs) across five stiff‐stalk assemblies. (a) Distribution of log_10_–transformed lengths for the four most common structural variants detected. (b) Number of SVs belonging to the four most common variant types. (c) Cumulative length of the four most common variant types

Structural variation between B73 and the five stiff‐stalk inbreds was primarily a result of genomic deletions, insertions, inversions, and duplications with sizes ranging from small insertions of 31 bp up to inversions as large as 6.14 Mbp (Figure 3a). The total number of SVs detected ranged from 23,197 in B84 to 42,295 in PHJ40. The number of SVs categorized as deletions or insertions was influenced by relatedness to the B73 comparator; lines such as B84, LH145, and NKH8431 had fewer SVs relative to PHB47 and PHJ40; however, the proportion of SVs categorized as deletions was consistent across the five stiff‐stalk inbreds (69.13–75.08%) (Figure 3b). In a genomic context, deletions were the predominant SV across all five stiff‐stalk inbreds, representing 197.74 Mbp (9.28%) of the B84 assembly to 447.60 Mbp (20.78%) in PHJ40, which was the most fragmented assembly. We noted an enrichment of deletions in the 9–11 kb size class (Figure 3a) and, upon inspection of TE annotations of deleted sequences, we found 4.6 times more fl‐LTRs, which are also typically in the 9–11 kb size range when compared with the random expectation (*p* < .00001, Fisher's exact test). Insertions represented 39–48 Mbp in four of the five stiff‐stalk inbreds, excepting PHJ40, which contained 97.65 Mbp of SVs categorized as insertions. Although few in number, inversions made up a substantial proportion of the stiff‐stalk nucleotide content (Figure 3c). Notably, LH145 contained 59.34 Mbp of inverted sequence (2.72% of the assembly), which was substantially greater than the other stiff‐stalk lines of which the next largest inversion content was 41.00 Mbp in line NKH8431 (1.93% of the assembly). The largest inverted region was found in NKH8431 (6.14 Mbp) on chromosome 4 at 96.76 Mbp. Duplicated SVs made up a small fraction of stiff‐stalk assemblies in terms of both number and nucleotide content.

### 3.5 Resistance gene diversity

Disease resistance genes are well documented as fast evolving gene families (Michelmore et al., 2013; Krattinger & Keller, 2016 ) and access to six stiff‐stalk genomes that arose through artificial selection provides a powerful dataset to understand the extent of diversity in a set of closely related genomes. The predicted proteomes of the six stiff‐stalk genomes were categorized into classes of resistance genes based on the detection of domains associated with disease resistance (Osuna‐Cruz et al., 2018). The six stiff‐stalk inbreds had similar putative resistance gene profiles (Supplemental Table S12); in total, 19 unique classes of resistance genes were identified in the predicted proteomes from the six stiff‐stalk inbreds. The six stiff‐stalk predicted proteomes contained similar quantities of putative resistance genes, ranging from 1,818 in B73 to 1,903 in LH145, with kinases and receptor‐like kinases representing approximately 49 and 27% of putative resistance genes, respectively. In comparison, proteins classified as receptor‐like kinases made up 42 and 36% of the putative resistance genes detected in sorghum and Arabidopsis, respectively.

The 1,818 predicted B73 resistance genes were compared across the stiff‐stalk inbreds. As disease resistance genes can share significant sequence similarity, we used synteny to determine the presence of syntelogs between B73 and the five stiff‐stalk inbreds. Of the 1,818 putative B73 resistance genes, only 202 (11%) were unique to B73 (Supplemental Figure S4). When a B73 resistance gene was present in at least one of the five stiff‐stalk inbreds, the most common copy number was four instead of the expected five. Both biological and technical factors are likely contributing to this value, since PHJ40 is more distantly related to B73, compared with the other lines, and also has a more fragmented assembly. Indeed, the number of resistance gene syntelogs to B73 ranged from 327 (PHJ40) to 1,485 (B84), representing 17–64% of the total syntelogs for their respective pairwise comparison. When PHJ40 was excluded from the analyses, the most common copy number was four, which corresponds to the number of non‐B73 stiff‐stalk inbreds. Some B73 resistance genes were duplicated in the stiff‐stalk genomes, most notably, a cluster of kinases near 188 Mbp on chromosome 1 (Supplemental Table S13); these B73 genes were annotated as wall‐associated kinases and were highly expressed in the leaf tissue.

**FIGURE 4:**
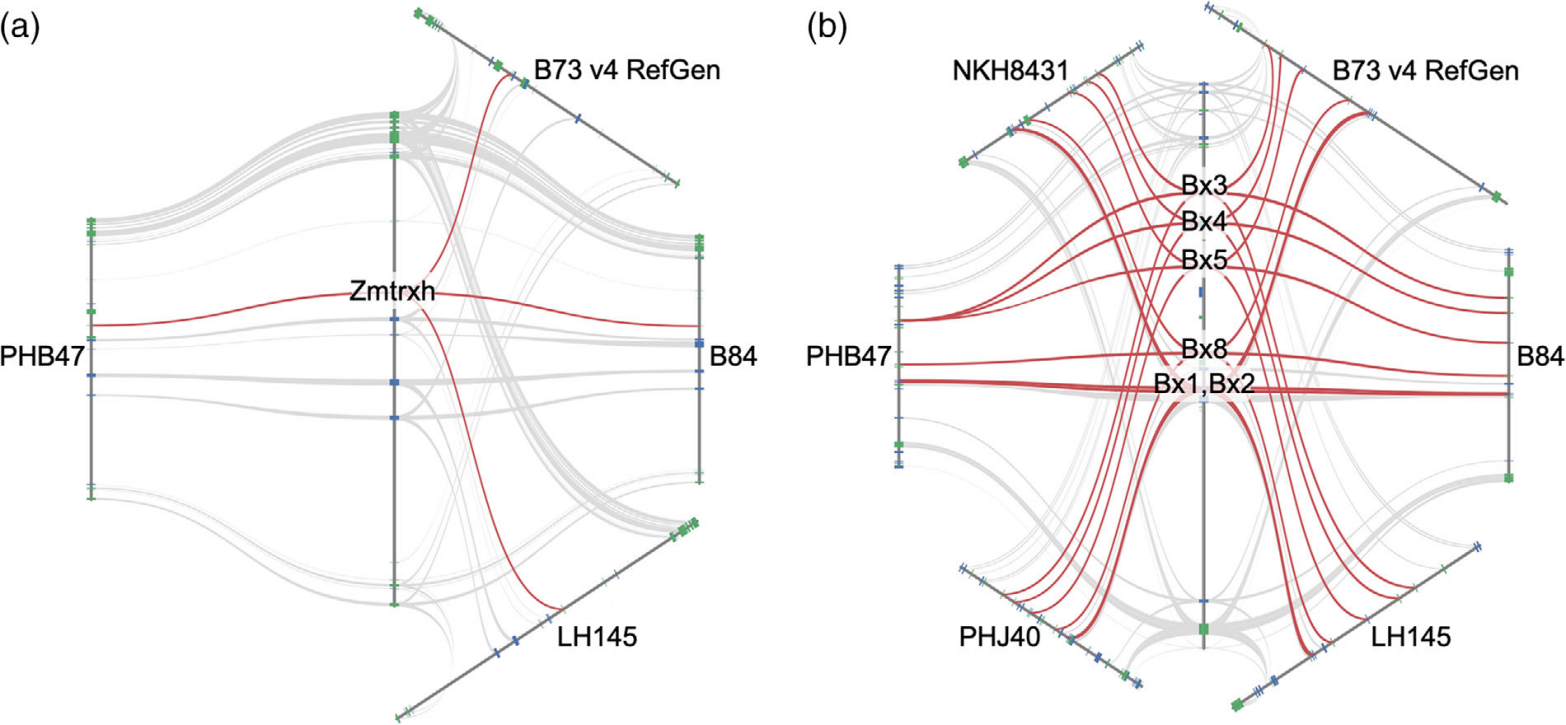
Resistance gene synteny among stiff‐stalk inbreds. Coding sequences of the stiff‐stalk inbreds were aligned to the B73 v4 MSU annotation and syntenic regions were visualized with the python version of MCscan (https://github.com/tanghaibao/jcvi/wiki/MCscan‐(Python‐version) implemented in the jcvi toolkit v1.1.7 with default parameters. (a) The stiff‐stalk inbreds exhibit presence–absence variation of the Zmtrxh locus near 24 Mbp on chromosome 6. (b) The stiff‐stalk inbreds exhibit complete conservation of DIMBOA gene cluster near 3.7 Mbp on chromosome 4. Relevant syntenic genes are highlighted by red connections, and adjacent syntenic genes are highlighted by grey connections. Genes on the forward and reverse strands are colored blue and green, respectively

Presence–absence variation has been well documented in maize (Springer et al., 2009; Lai et al., 2010; Hirsch et al., 2014, 2016). To highlight this phenomenon, we investigated a previously characterized gene conferring resistance to sugarcane mosaic virus, *ZmTrxh* (Liu et al., 2017). This gene is located on chromosome 6 near 24 Mbp in the B73 inbred line and is within a known PAV (Gustafson et al., 2018; Gage et al., 2019). *ZmTrxh* was present in a large collinear block shared among B73 and three stiff‐stalk inbreds: B84, LH145, and PHB47 (Figure 4a). When the B73 ZmTrxh protein sequence was queried against the six stiff‐stalk genomes, no hits were detected in the NKH8431 and PHJ40 genome assemblies, suggesting that it is a PAV in these two inbreds. Previous disease incidence scores indicate that SCMV resistance is quantitative, and that presence of *Scmv1* within the PAV is necessary but not sufficient for SCMV resistance (Gustafson et al., 2018). In contrast, a cluster of genes encoding the biosynthesis of DIMBOA (2,4‐dihydroxy‐7‐methoxy‐1,4‐benzoxazin‐3‐one) near 3.7 Mbp on chromosome four were completely conserved across all stiff‐stalk inbreds (Figure 4b). These findings further support the notion that general defense mechanisms, such as DIMBOA biosynthesis conferring broad resistance across plant pathogens, are more highly conserved than single‐gene‐based disease resistance.

### 3.6 Founders and conserved regions in descendants

**FIGURE 5:**
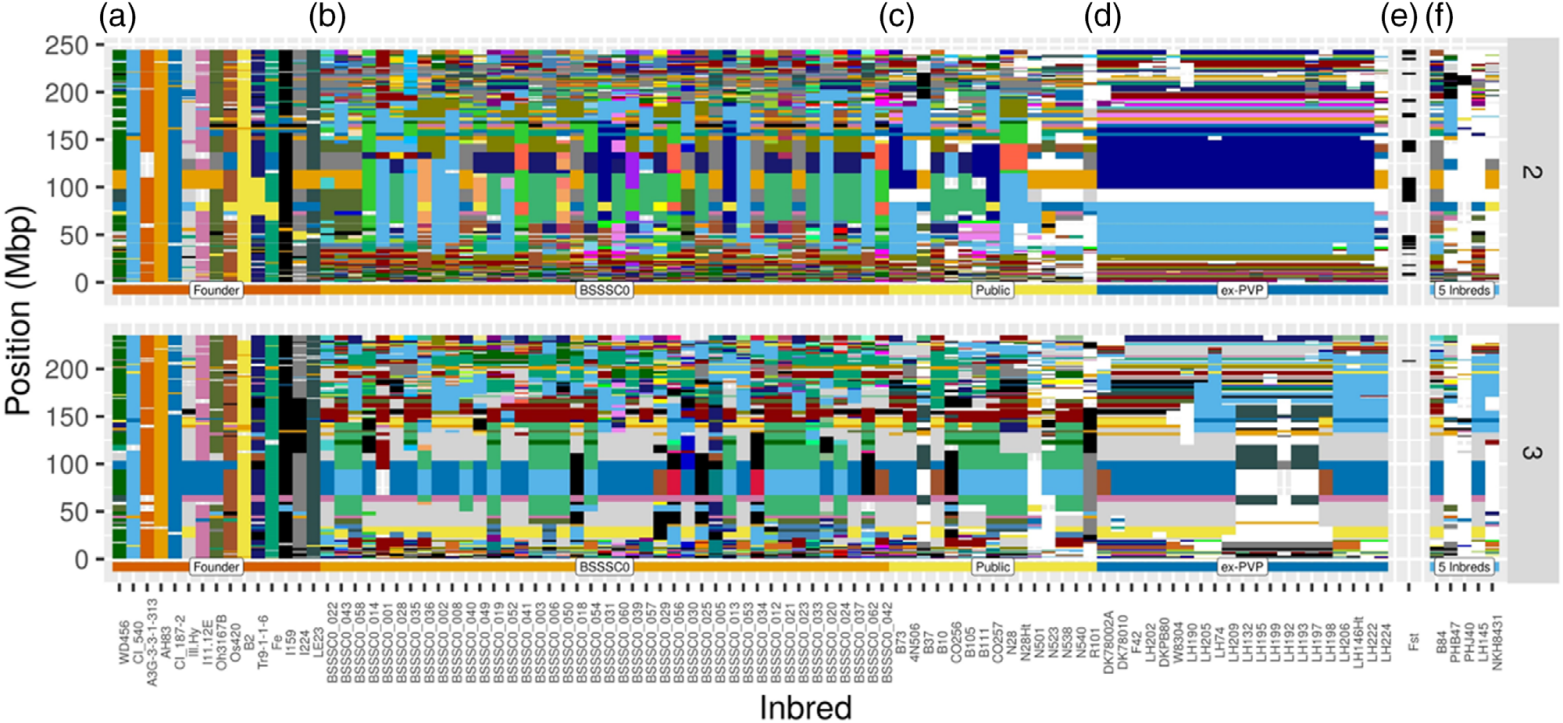
Stiff‐stalk haplotypes and block *F*_ST_ values. (a,b) 960‐SNP window haplotype blocks for founder inbreds (a) and unselected BSSSC0 inbreds (b) for chromosomes 2 and 3. (c,d) Conserved BSSS haplotypes for public releases from the BSSS populations (c) and highly related ex‐PVP inbreds (d). Haplotypes not found in the founders or BSSSC0 lines are plotted in white. (e) Black boxes indicate haplotypes with binned maximum *F*_ST_ values in the top 10th percentile of genome‐wide binned *F*_ST_ values. The *F*_ST_ value was calculated between the unselected lines, composed of the Founder and BSSSC0 inbreds, and selected lines, composed of the Public and ex‐PVP inbreds. (f) Founder and BSSSC0 haplotypes present in the five assembled inbreds. Haplotypes with missing data are not plotted, showing the background of the plot. Major commercial inbred name prefixes: LH, Holden's Foundation Seeds, now owned by Bayer; DK, DeKalb Genetics Corporation, now owned by Bayer; PH, Pioneer Hi‐Bred International, now owned by Corteva. For full descriptions of inbreds, see the Germplasm Resource Information Network database or (Mazaheri et al., 2019). BSSS, Iowa Stiff Stalk Synthetic; ex‐PVP, expired Plant Variety Protection

We sought to determine the representation of the BSSS population within the five stiff‐stalk inbreds evaluated and a group of publicly released or commercial ex‐PVP inbreds. B84 is directly from BSSS (HT)C7, and the four ex‐PVP lines have one or more inbreds in their lineage derived directly from a version of BSSS. The founder inbreds are diverse amongst themselves, having only a few small regions that are shared by more than two lines, as exemplified by the founder haplotypes on chromosomes two and three (Figure 5a; remaining chromosomes in Supplemental Figures S5 and S6). Likewise, BSSSC0 inbreds show a mosaic of shared haplotypes with the founders on chromosomes 2 and 3 and exhibit much shorter contiguous haplotypes, as expected after several generations of recombination and inbreeding (Figure 5b). Two founder lines are absent from our analysis, resulting in some BSSSC0 lines containing haplotypes that are not present in the founder lines. For the publicly released and ex‐PVP inbreds, haplotypes that are not found in the base BSSS population are plotted in white to facilitate visualization of BSSS haplotype conservation. The public inbreds have a greater diversity of haplotypes present than the ex‐PVP inbreds, which exhibit a large reduction in diversity potentially a result of the founder effects of commercial usage of B73 (Figure 5c, 5d). Haplotype blocks are largest, as measured in distance in base pairs, in pericentromeric regions, which is expected because of lower SNP density in the RNA‐seq data and lower levels of recombination (Figure 5a). Several haplotypes move to fixation on both chromosome 2 and chromosome 3, but only chromosome 2 shows significantly elevated *F*_ST_ compared with the genome‐wide average. Twenty‐four out of 109 blocks on chromosome 2 rank in the highest 10th percentile of genome‐wide *F*_ST_ values, having a value >0.53, while only one out of 101 blocks on chromosome three ranks with high *F*_ST_ (Figure 5e; Supplemental Table S14).

In Figure 5f, BSSS founder and BSSSC0 haplotypes are plotted for the five assembled stiff‐stalk genomes. As in the publicly released and ex‐PVP lines, non‐BSSS haplotypes are plotted in white. As expected, B84 has high levels of conservation of the base BSSS population. Of the 900 total genome‐wide blocks, 87.1% of blocks in B84 are from the founder or BSSSC0 lines. LH145 shares 64.7%, NKH8431 shares 57.6%, PHB47 shares 67.8%, and PHJ40 shares the least haplotype blocks with the base BSSS population at 29% (data not shown). There are several possible reasons for the haplotype blocks that are unique to B84 compared with the base BSSS population. In addition to the absence of two founder lines from our study, the unique haplotypes could be due to genotyping error, residual heterozygosity, mutation, or population contamination sometime during development or maintenance of the line. A range of 12.9 (B84) to 71% (PHJ40) of the genome‐wide haplotype blocks in the sequenced inbreds come from outside the base BSSS population, as demonstrated by the white segments in Figure 5f, which highlights the unique and diverse nature of these five lines despite their common placement in the stiff‐stalk heterotic pool.

Genome‐wide IBS was calculated for each of the five lines with their respective closest stiff‐stalk founders. As expected, PHB47 has a high level of identity with its parent B37, where 73.1% of the 900 genome‐wide haplotype windows have >97% IBS. Despite this high level of IBS, identity is not distributed evenly in the genome, and seven of 10 centromere‐containing regions are diverse between the two lines (Supplemental Figure S7). LH145 has high identity with its founder B14, which is found in the backgrounds of both of its parents, A632Ht and CM105. The pedigree of A632 (sans “Ht”, Northern Corn Leaf Blight resistance) is B14 crossed to Mt42 with three backcrosses to B14, and B14 is also a direct parent to CM105 (npgsweb.ars‐grin.gov). LH145 and B14 have high IBS in 64.4% of genome wide windows (Supplemental Figure S8). B84 shares 39.0% of IBS windows with B73 and has fewer and shorter conserved haplotypes than the direct relationship of PHB47 with B37 and LH145 with B14 (Supplemental Figure S9). NKH8431 has a higher level of IBS with B14 at 40.9% window sharing than B73 at 25.8%, which is expected because of its pedigree that includes two parents derived from B14 and one parent derived from B73 (Supplemental Figures S10 and S11). Finally, PHJ40 has IBS >97% in 24.3% of genome windows with B37, with conserved haplotypes that are concentrated on chromosomes 1, 4, and 9 (Supplemental Figure S12). The ancestral pedigrees of the proprietary inbreds used to generate PHJ40 are not known, but previous work indicates that B37 is a contributor to PHJ40, with minor admixture from Lancaster and Oh43 germplasm (White et al., 2020). The lower level of IBS between B37 and PHJ40 is consistent with previous observations in this study that PHJ40 is more distantly related than the other stiff‐stalk inbreds and agrees with our findings as well (Figure 1a; Supplemental Tables S8 and S9).

As B73 is considered the reference genome for the maize community, we examined the relationship between SV and IBS regions in detail. Structural variants between B73 and B84 >100,000 bp, including insertions, deletions, and inversions, were plotted for each chromosome (Supplemental Figure S9). Increased SV density was associated with decreased SNP IBS as expected. Some regions with long stretches of high IBS do contain SVs, which could be due to the method of generating the SNPs by aligning RNA‐Seq reads to the B73 reference, or decreased SNP density, such that the consecutive conserved SNPs fall on either side of the SV. Overall, SVs between B73 and B84 occur in nonconserved regions between the two lines.

Finally, we sought to determine the proportions of stiff‐stalk founders B14 and B37 that were present within the five stiff‐stalk inbreds that we sequenced. As previously noted, inbreds LH145, NKH8431, and B84 have direct relationships with B14, and 85.6% of the 900 genome‐wide windows have IBS >0.97 between B14 and any of its related inbreds (Supplemental Figure S13). Similarly, 81.6% of the genome‐wide windows are conserved between B37 and its related inbreds PHB47, PHJ40, and B84 (Supplemental Figure S14). Thus, a high proportion of the genomic sequence of stiff‐stalk founders B14 and B37 is represented in the inbreds sequenced in this study.

## 4 CONCLUSIONS

Here we provide genomic resources for five historically important commercial stiff‐stalk inbred lines. High‐quality de novo genome assemblies were generated with PacBio long‐read sequencing that contain near‐complete coverage of genic space as well as substantial repetitive content supporting the high‐quality nature of the assemblies. Inbred‐specific transcriptomes and gene annotations were independently generated using a core set of five tissues that permitted unconfounded comparisons of gene content across six key stiff‐stalk inbreds revealing broad similarity yet unique regions, reaffirming their usefulness in heterotic pattern breeding schemes.

The stiff‐stalk population has been an important source of seed parent germplasm for maize breeders in the public and private sectors since the mid 20th century. It is estimated that B14, B37, and B73 have an overall genetic contribution of 3.2, 1.5, and 11.7%, respectively, to inbred lines registered between 2004 and 2008 by the commercial breeding programs of Monsanto (now Bayer), PHI (now Corteva), and Syngenta (Mikel, 2011). A study of ex‐PVP inbreds estimated admixture of recently developed lines through kinship analysis and found that of the 1,506 lines with kinship estimates, developed in the year 2000 or later, 15% had total stiff‐stalk admixture >50%, and 33% of lines had stiff‐stalk admixture >30% (White et al., 2020). Reciprocal recurrent selection in maize breeding has increased genetic distance between the stiff‐stalk and non‐stiff‐stalk groups, as exemplified by increasing distance between the progressive cycles of BSSS and its partner population, the Iowa Corn Borer Synthetic No.1 (Hinze et al., 2005). Complementation of deleterious, incompletely dominant alleles has been previously shown to drive hybrid vigor between heterotic groups (J. Yang et al., 2017). Thus, selection for heterotic hybrids in the stiff‐stalk by non‐stiff‐stalk overall heterotic groups would be expected to drive divergent allele frequency between groups and reduce allelic variation within groups. Our results support that released inbreds, especially ex‐PVP, contain quite limited allelic variation compared with that present in the original BSSS population, as represented by random BSSSC0 and founder inbreds in this study. Drift has previously been shown to play a major role in the population structure of the BSSS and the Iowa Corn Borer Synthetic No.1 (Gerke et al., 2015). Drift and founder effects likely contribute to the fixation of haplotypes that we observe, yet the fixation of rare haplotypes can contribute to genetic gain and phenotypic improvement if they contain favorable alleles for yield, heterosis, disease resistance, or agronomic improvement. As examples of changes observed through selection and drift, the combination of haplotypes spanning ∼200 Mbp on chromosome 2 present in B73 did not exist in the base BSSS population but reached fixation within a group of commercial germplasm, while a common haplotype present within the BSSS founders on chromosome 3 did not reach total fixation. Genetic diversity is vital to continued genetic improvement, and our results support that substantial genetic diversity remains within the broadly defined stiff‐stalk heterotic pool. Empirical studies also indicate that yield heterosis may be found in noncanonical hybrids produced from inbreds from different stiff‐stalk subgroups. In a diallel of 13 inbreds from different heterotic patterns, hybrids PHB47 × NKH8431 and PHB47 × LH145 had the highest specific combining ability, suggesting that sufficient genetic diversity exists between the stiff‐stalk subgroups to form competitive hybrids and certainly produce phenotypic segregation in crosses (White et al., 2020).

Founder haplotype conservation is demonstrated in each of the five stiff‐stalk inbreds assessed in this study. Selection on the BSSS population by Iowa State University followed by incorporation into commercial breeding programs has led to the accumulation of alleles potentially important for yield and agronomic traits. These five stiff‐stalk inbreds represent founder alleles in elite contexts, which can aid the maize genetics community in the study of yield, quantitative traits, and adaptation to variable environments. In addition, the five stiff‐stalk inbreds span the genetic and institutional diversity of the pool, representing both heterotic subgroups and North American maize breeding entities in the 1980s, including Iowa State University, PHI, Holden's Foundation Seeds, and Northrup King. Thus, these lines can be used to study the population of alleles present within the stiff‐stalk heterotic group that contribute to adaptation, genotype‐by‐environment interactions, and combining ability between the stiff‐stalk subgroups and non‐stiff‐stalk subgroups. Substantial genetic and genomic diversity was identified within the assembled inbreds despite their highly selected and adapted nature, and diversity likely remains within the greater stiff‐stalk pool to be explored and used by maize breeders and geneticists.

## AUTHOR CONTRIBUTIONS

K.B., C.D., J.G., H.H., J.J., M.K., C.P., J.S., S.S., V.S., J.T., and Y.Y. generated data. N.B., K.M., J.P.H., S.O., A.S.S., and J.J. analyzed data. N.dL., S.M.K., and C.R.B. conceived of the study. N.B., K.M., J.P.H., S.O., and C.R.B. wrote the manuscript. J.S., C.N.H, M.B.H., N.dL., S.M.K., and C.R.B. supervised the work.

## CONFLICT OF INTEREST

The authors declare no conflict of interest.

## Supporting information

Supplemental Figure S1. Distribution of LTR Assembly Index (LAI) values.
Supplemental Figure S2. Summary of LTR Assembly Index (LAI) values.
Supplemental Figure S3. Distribution of insertion time of intact LTR retrotransposons.
Supplemental Figure S4. Resistance genes in syntenic blocks.
Supplemental Figure S5. Stiff‐stalk haplotype and block Fst values for chromosomes one, four, and five.
Supplemental Figure S6. Stiff‐stalk haplotype and block Fst values for chromosomes six through ten.
Supplemental Figure S7. Chromosomal IBS of PHB47 and B37.
Supplemental Figure S8. Chromosomal IBS of LH145 and B14.
Supplemental Figure S9. Chromosomal IBS of B84 and B73.
Supplemental Figure S10. Chromosomal IBS of NKH8431 and B14.
Supplemental Figure S11. Chromosomal IBS of NKH8431 and B73.
Supplemental Figure S12. Chromosomal IBS of PHJ40 and B37.
Supplemental Figure S13. Chromosomal conservation of B14.
Supplemental Figure S14. Chromosomal conservation of B37.

Supplemental Table S1. Construction of the scaffold assemblies of five stiff‐stalk inbred genomes

Supplemental Table S2. Whole‐genome sequencing read mapping statistics for six stiff‐stalk inbreds genome assemblies.

Supplemental Table S3. RNA‐Seq read mapping statistics for six stiff‐stalk inbred genome assemblies.

Supplemental Table S4. BUSCO metrics for 6 stiff‐stalk genome assemblies and annotations.

Supplemental Table S5. Summary of the LTR Assembly Index for six stiff‐stalk inbred genomes.

Supplemental Table S6. Percentage of genome assembly annotated as a repetitive sequence.

Supplemental Table S7. Panorthologous group and protein metrics for six stiff‐stalk inbreds.

Supplemental Table S8. Proportion of high confidence representative coding sequence present in each assembly.

Supplemental Table S9. Pangene groupings for six stiff‐stalk inbreds.

Supplemental Table S10. Pangene groupings for six stiff‐stalk inbreds, PH207, and Mo17.

Supplemental Table S11. Summary of MCscanX collinear blocks for six stiff‐stalk inbred genomes.

Supplemental Table S12. Disease resistance genes in the predicted proteomes of six maize stiff‐stalks, *Sorghum bicolor*, and *Arabidopsis thaliana*.

Supplemental Table S13. Duplicated DRAGO2 syntelogs on chromosome 1.

Supplemental Table S14. Genome‐wide window *F*_ST_ values and haplotype block colors.

## Data Availability

Raw genome sequence reads are available in the NCBI Sequence Read Archive under the BioProject identifiers listed in Supplemental Tables S1 and S2. The genome assemblies have been deposited in NCBI under accession numbers B84 (JAGTWB000000000), LH145 (JAGTWC000000000), NKH8431 (JAGTWD000000000), PHB47 (JAGTWE000000000), and PHJ40 (JAGTWF000000000). RNA‐seq reads used in this study were obtained from the NCBI Sequence Read Archive samples listed in Supplemental Table S3. The genome assemblies and associated annotation for all stiff‐stalk inbreds described in this study (B73, B84, LH145, NKH8431, PHB47, and PHJ40) are available from the Maize Genomics Resource at Michigan State University (http://maize.plantbiology.msu.edu/) via a genome browser, BLAST search tool, and flat files. Large data files have been deposited at the Dryad Digital Repository (https://doi.org/10.5061/dryad.wh70rxwmw). The repository includes genome assembly, genome annotation, expression abundances, syntenic blocks, orthologous groups, resistance genes, and pangenome membership.
