# Supplementary material for: Genomic variation within the maize stiff‐stalk heterotic germplasm pool
Source: Plant Genome. 2021 Jul 18;14(3):e20114. doi: 10.1002/tpg2.20114 (PMC12806955; doi:10.1002/tpg2.20114)
Supplement: Supplementary file 1 [file TPG2-14-e20114-s009.pdf]

Supplemental Information for

Genomic variation within the maize Stiff Stalk heterotic germplasm pool

Nolan Bornowski[1]*, Kathryn J. Michel[2]*, John P. Hamilton[1], Shujun Ou[3], Arun S. Seetharam[3], Jerry Jenkins[4], Jane Grimwood[4], Chris Plott[4], Shengqiang Shu[5], Jayson Talag[6], Megan Kennedy[5], Hope Hundley[5], Vasanth R. Singan[5], Kerrie Barry[5], Chris Daum[7], Yuko Yoshinaga[7], Jeremy Schmutz[4,5], Candice N. Hirsch[7], Matthew B. Hufford[3], Natalia de Leon[2,8], Shawn M. Kaeppler[2,8,9], C. Robin Buell[1,10]

[1] Department of Plant Biology, Michigan State University, 612 Wilson Road, East Lansing, MI 48824
[2] Department of Agronomy, University of Wisconsin – Madison, 1575 Linden Drive, Madison, WI 53706
[3] Department of Ecology, Evolution, and Organismal Biology, Iowa State University, 2200 Osborn Drive, Ames, IA 50011
[4] HudsonAlpha Institute for Biotechnology, 601 Genome Way Northwest, Huntsville, AL 35806
[5] U.S. Department of Energy Joint Genome Institute, Lawrence Berkeley National Laboratory, 1 Cyclotron Road, Berkeley, CA 94720
[6] Arizona Genomics Institute, School of Plant Sciences, University of Arizona, 1657 E Helen Street, Tucson, AZ 85721
[7] Department of Agronomy and Plant Genetics, University of Minnesota, 1991 Upper Buford Circle, Saint Paul, MN 55108
[8] Department of Energy Great Lakes Bioenergy Research Center, University of Wisconsin – Madison, 1575 Linden Drive, Madison, WI 53706
[9] Wisconsin Crop Innovation Center, University of Wisconsin – Madison, 8520 University Green, Middleton, WI 53562
[10] Department of Energy Great Lakes Bioenergy Research Center, Michigan State University, 612 Wilson Road, East Lansing, MI 48824

Corresponding author: buell@msu.edu

This PDF file contains:

Supplemental Figure Legends
Supplemental Figures
Supplemental Table Titles
References

Other supporting materials for this manuscript include the following:
Supplemental Tables
DataDryad repository doi:10.5061/dryad.wh70rxwmw

Supplemental Figure Legends

Supplemental Figure S1. Distribution of LTR Assembly Index (LAI) values.
Values were estimated on 3 Mbp sliding windows with a step size of 300 kbp. Blue dotted lines show the genome-wide LAI.

Supplemental Figure S2. Summary of LTR Assembly Index (LAI) values.
Values were estimated on 3 Mbp sliding windows with a step size of 300 kbp. Left boxes (salmon) are LAI of the scaffolded regions (pseudomolecules). Right boxes (teal) are LAI of the unplaced scaffolds. The box shows the median, upper, and lower quartiles. Whiskers indicate values ≤ 1.5× of the interquartile range. Black dots indicate outliers.

Supplemental Figure S3. Distribution of insertion time of intact LTR retrotransposons.
The insertion time of each element was estimated with $T = K/2\mu$ where K is the divergence between the left and right LTR of the element and $\mu = 3.3e\text{-}8$ per bp per year for heterochromatic regions (Clark et al., 2005).

Supplemental Figure S4. Resistance genes in syntenic blocks.
Putative B73 resistance genes were characterized according to domains associated with disease resistance. These putative resistance genes were queried against the other five Stiff Stalks to identify syntelogs occurring on the same chromosome and similar genomic location. (a) Frequency of predicted B73 resistance genes among five Stiff Stalks. A total of 202 B73 genes did not have a syntelog, while 49% of predicted B73 resistance genes had four copies among the five Stiff Stalks. (b) Upset plot (Conway et al., 2017) of the predicted B73 resistance gene syntelog presence/absence variants among five Stiff Stalk genomes. Syntelogs present in four of the five Stiff Stalks were most frequent due to PHJ40 only containing syntelogs for 322 B73 genes. In contrast, B84 contained syntelogs for 1470 B73 genes. Unique syntelogs between each of the five Stiff Stalks and the B73 predicted resistance genes are highlighted in the upset plot. (c) Frequency of predicted B73 resistance genes among four Stiff Stalks (PHJ40 excluded). (d) Upset plot of the predicted B73 resistance gene syntelog presence/absence variants among four Stiff Stalk genomes (PHJ40 excluded).

Supplemental Figure S5. Stiff Stalk haplotype and block Fst values for chromosomes one, four, and five.
(a,b) 960 SNP window haplotype blocks for founder inbreds (a) and unselected BSSSC0 inbreds (b) for chromosomes one, four, and five. (c,d) Conserved BSSS haplotypes for public releases from the BSSS populations (c) and highly related ex-PVP inbreds (d). Haplotypes not found in the founders or BSSSC0 lines are plotted in white. (e) Black boxes indicate haplotypes with binned maximum Fst values in the top 10th percentile of genome-wide binned Fst values. Fst was calculated between the unselected lines, composed of the Founder and BSSSC0 inbreds, and selected lines, composed of the Public and ex-PVP inbreds. (f) Founder and BSSSC0 haplotypes present in the five assembled inbreds. Haplotypes with missing data are not plotted, showing the background of the plot. Major commercial inbred name prefixes: LH (Holden's Foundation Seeds, now owned by Bayer), DK (DeKalb Genetics Corporation, now owned by

Bayer), PH (Pioneer Hi-Bred International, now owned by Corteva). For full descriptions of inbreds, see the Germplasm Resource Information Network database or (Mazaheri et al., 2019).

Supplemental Figure S6. Stiff Stalk haplotype and block Fst values for chromosomes six through ten.
(a,b) 960 SNP window haplotype blocks for founder inbreds (a) and unselected BSSSC0 inbreds (b) for chromosomes six through ten. (c,d) Conserved BSSS haplotypes for public releases from the BSSS populations (c) and highly related ex-PVP inbreds (d). Haplotypes not found in the founders or BSSSC0 lines are plotted in white. (e) Black boxes indicate haplotypes with binned maximum Fst values in the top 10th percentile of genome-wide binned Fst values. Fst was calculated between the unselected lines, composed of the Founder and BSSSC0 inbreds, and selected lines, composed of the Public and ex-PVP inbreds. (f) Founder and BSSSC0 haplotypes present in the five assembled inbreds. Haplotypes with missing data are not plotted, showing the background of the plot. Major commercial inbred name prefixes: LH (Holden's Foundation Seeds, now owned by Bayer), DK (DeKalb Genetics Corporation, now owned by Bayer), PH (Pioneer Hi-Bred International, now owned by Corteva). For full descriptions of inbreds, see the Germplasm Resource Information Network database or (Mazaheri et al., 2019).

Supplemental Figure S7. Chromosomal IBS of PHB47 and B37.
Haplotype window mean identity by state values between PHB47 and B37 are plotted for each of the ten chromosomes, with vertical lines representing the approximate centromere position.

Supplemental Figure S8. Chromosomal IBS of LH145 and B14.
Haplotype window mean identity by state values between LH145 and B14 are plotted for each of the ten chromosomes, with vertical lines representing the approximate centromere position.

Supplemental Figure S9. Chromosomal IBS of B84 and B73.
Haplotype window mean identity by state values between B84 and B73 are plotted for each of the ten chromosomes, with vertical lines representing the approximate centromere position. Locations and lengths of deletions (DEL), insertions (INS) and inversions (INV) are noted as bubbles below the line graph.

Supplemental Figure S10. Chromosomal IBS of NKH8431 and B14.
Haplotype window mean identity by state values between NKH8431 and B14 are plotted for each of the ten chromosomes, with vertical lines representing the approximate centromere position.

Supplemental Figure S11. Chromosomal IBS of NKH8431 and B73.
Haplotype window mean identity by state values between NKH8431 and B73 are plotted for each of the ten chromosomes, with vertical lines representing the approximate centromere position.

Supplemental Figure S12. Chromosomal IBS of PHJ40 and B37.

Haplotype window mean identity by state values between PHJ40 and B37 are plotted for each of the ten chromosomes, with vertical lines representing the approximate centromere position.

Supplemental Figure S13. Chromosomal conservation of B14.
Haplotype window mean identity by state values between B14 and its descendants, LH145 and NKH8431, or related inbred B84 are plotted for each of the ten chromosomes, with vertical lines representing the approximate centromere position.

Supplemental Figure S14. Chromosomal conservation of B37.
Haplotype window mean identity by state values between B37 and its descendants, PHB47 and PHJ40, or related inbred B84 are plotted for each of the ten chromosomes, with vertical lines representing the approximate centromere position.

Supplemental Figures

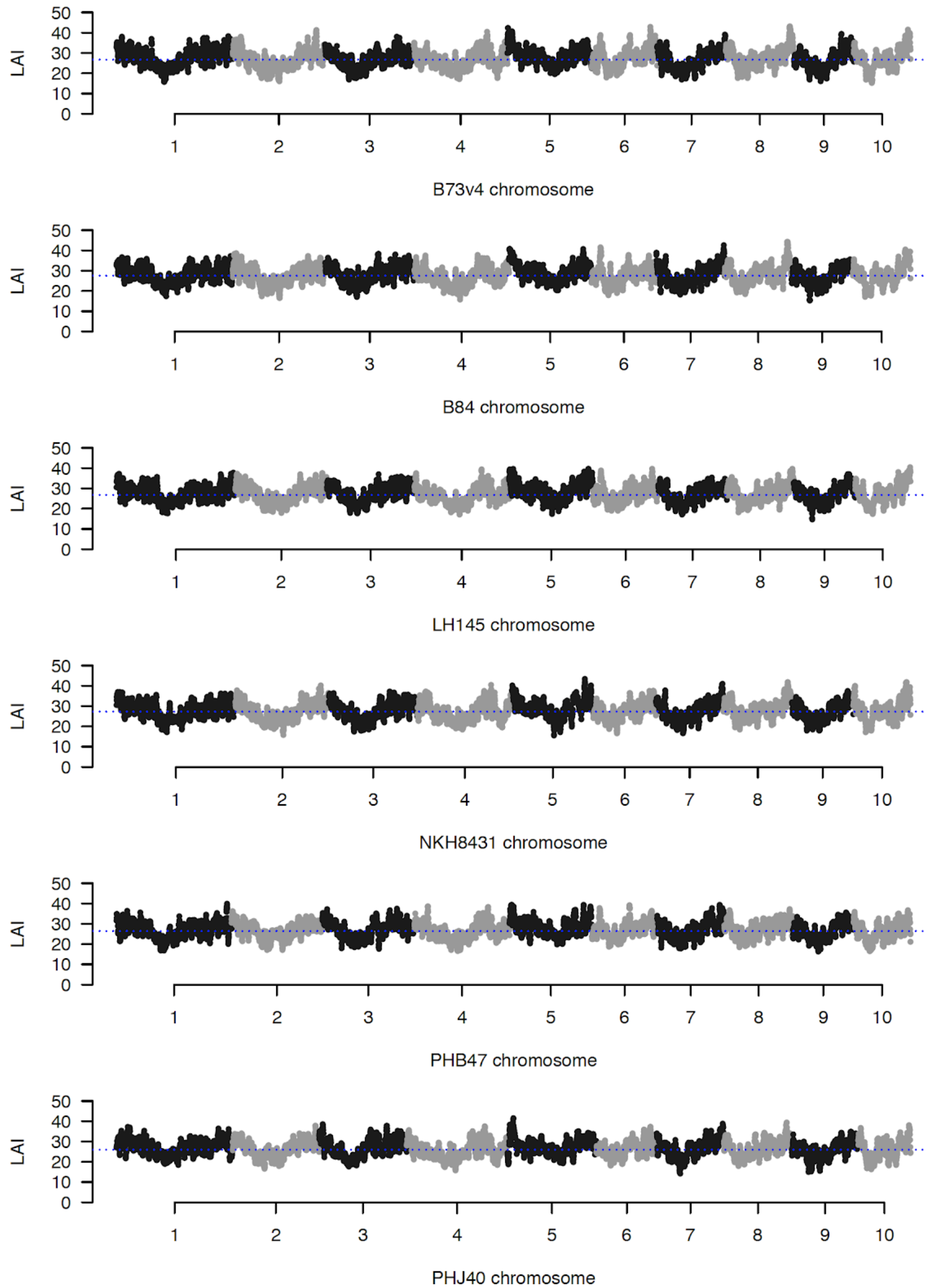

Supplemental Figure S1. Distribution of LTR Assembly Index (LAI) values. Values were estimated on 3 Mbp sliding windows with a step size of 300 kbp. Blue dotted lines show the genome-wide LAI.

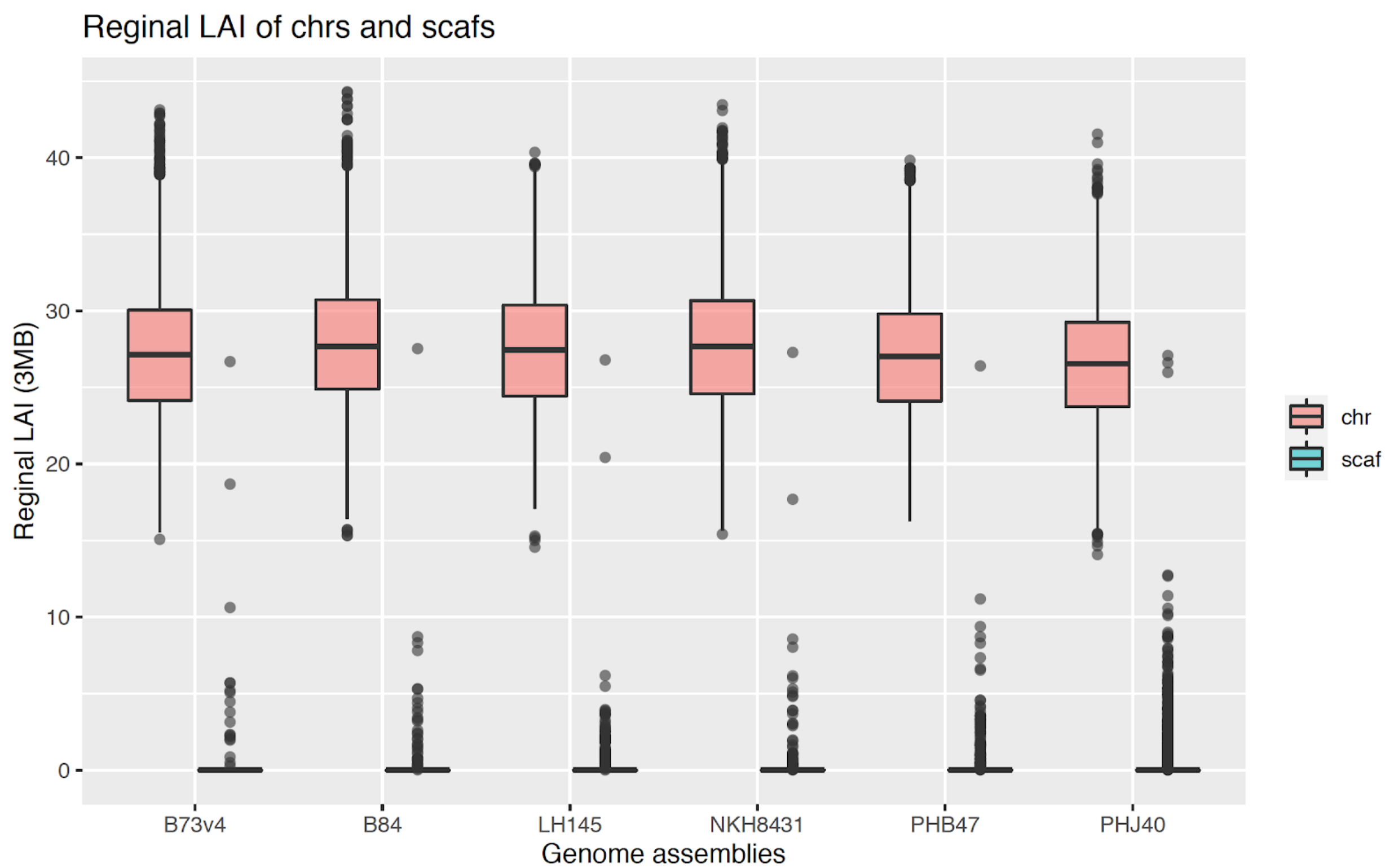

Supplemental Figure S2. Summary of LTR Assembly Index (LAI) values. Values were estimated on 3 Mbp sliding windows with a step size of 300 kbp. Left boxes (salmon) are LAI of the scaffolded regions (pseudomolecules). Right boxes (teal) are LAI of the unplaced scaffolds. The box shows the median, upper, and lower quartiles. Whiskers indicate values ≤ 1.5× of the interquartile range. Black dots indicate outliers.

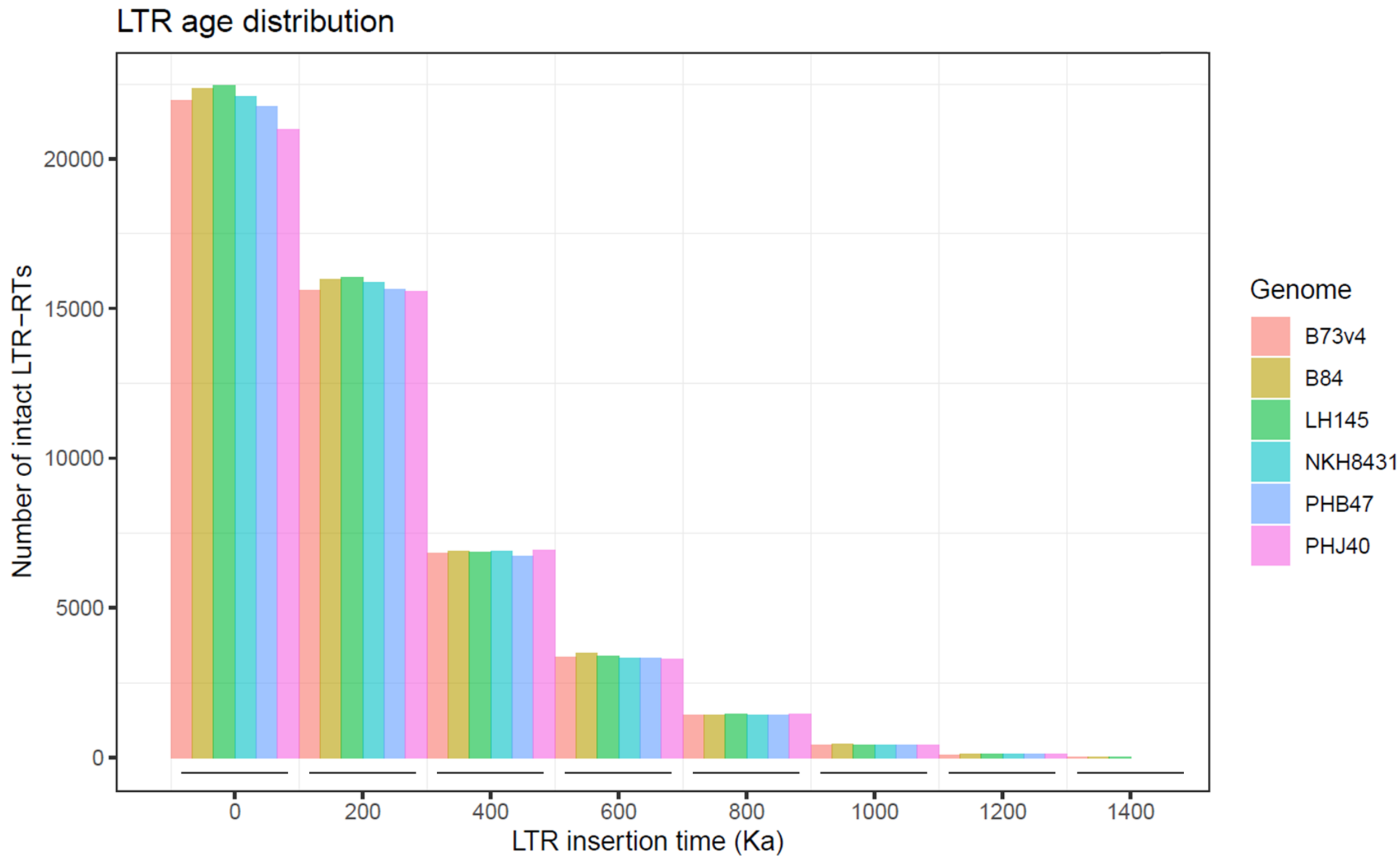

Supplemental Figure S3. Distribution of insertion time of intact LTR retrotransposons. The insertion time of each element was estimated with T = K/2µ where K is the divergence between the left and right LTR of the element and µ = 3.3e-8 per bp per year for heterochromatic regions (Clark et al., 2005).

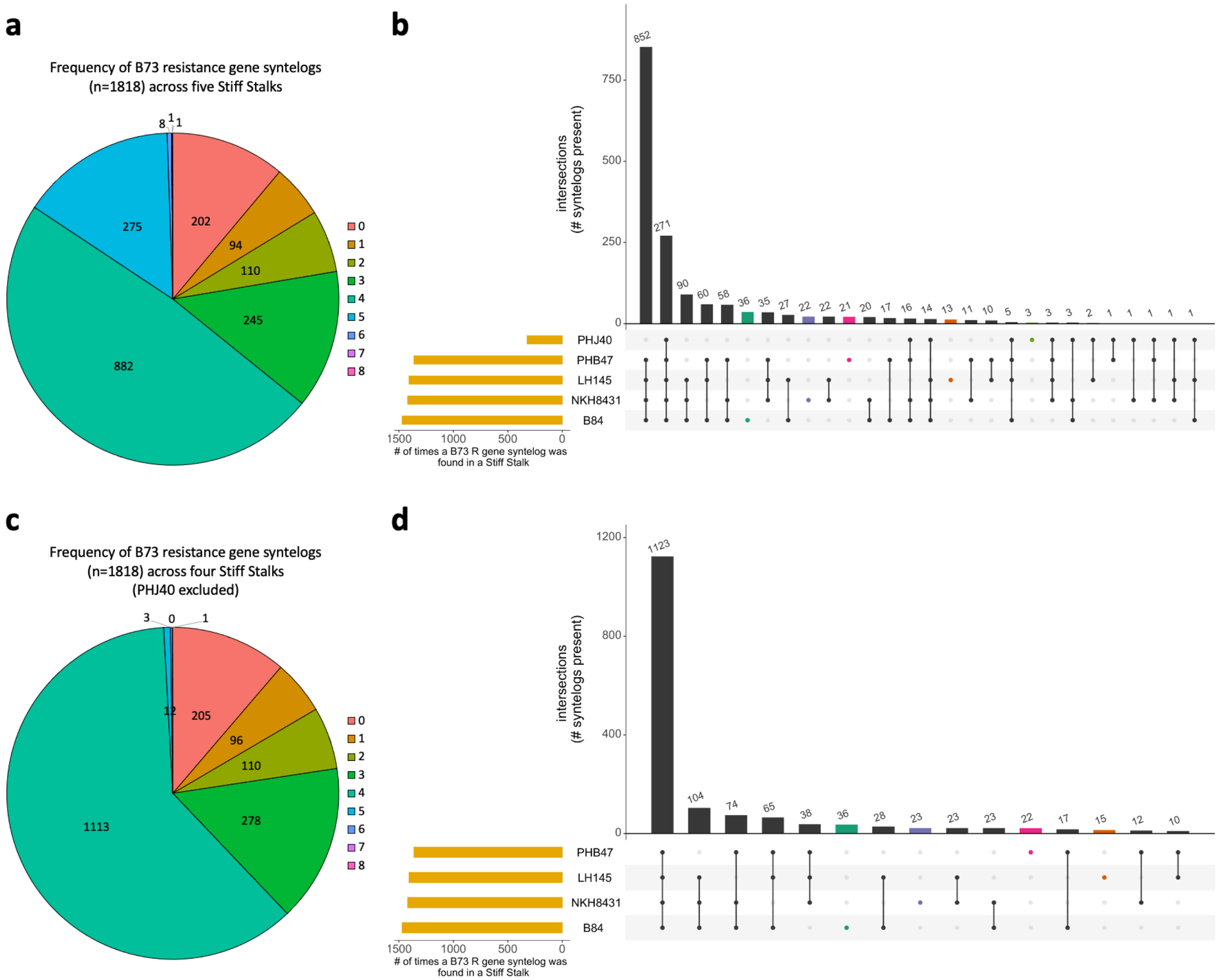

Supplemental Figure S4. Resistance genes in syntenic blocks. Putative B73 resistance genes were characterized according to domains associated with disease resistance. These putative resistance genes were queried against the other five Stiff Stalks to identify syntelogs occurring on the same chromosome and similar genomic location. (a) Frequency of predicted B73 resistance genes among five Stiff Stalks. A total of 202 B73 genes did not have a syntelog, while 49% of predicted B73 resistance genes had four copies among the five Stiff Stalks. (b) Upset plot (Conway et al., 2017) of the predicted B73 resistance gene syntelog presence/absence variants among five Stiff Stalk genomes. Syntelogs present in four of the five Stiff Stalks were most frequent due to PHJ40 only containing syntelogs for 322 B73 genes. In contrast, B84 contained syntelogs for 1470 B73 genes. Unique syntelogs between each of the five Stiff Stalks and the B73 predicted resistance genes are highlighted in the upset plot. (c) Frequency of predicted B73 resistance genes among four Stiff Stalks (PHJ40 excluded). (d) Upset plot of the predicted B73 resistance gene syntelog presence/absence variants among four Stiff Stalk genomes (PHJ40 excluded).

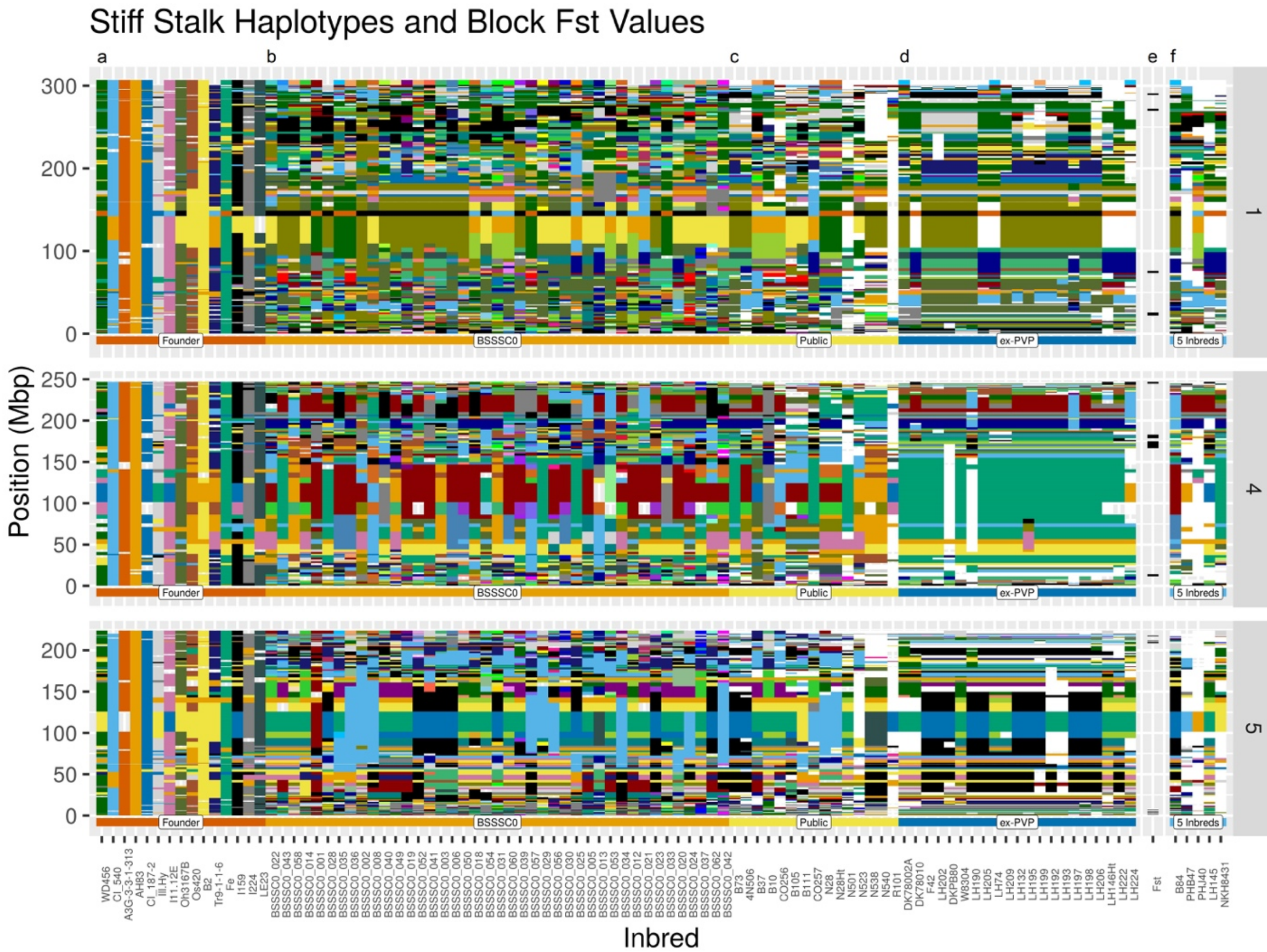

Supplemental Figure S5. Stiff Stalk haplotype and block Fst values for chromosomes one, four, and five. (a,b) 960 SNP window haplotype blocks for founder inbreds (a) and unselected BSSSC0 inbreds (b) for chromosomes one, four, and five. (c,d) Conserved BSSS haplotypes for public releases from the BSSS populations (c) and highly related ex-PVP inbreds (d). Haplotypes not found in the founders or BSSSC0 lines are plotted in white. (e) Black boxes indicate haplotypes with binned maximum Fst values in the top 10th percentile of genome-wide binned Fst values. Fst was calculated between the unselected lines, composed of the Founder and BSSSC0 inbreds, and selected lines, composed of the Public and ex-PVP inbreds. (f) Founder and BSSSC0 haplotypes present in the five assembled inbreds. Haplotypes with missing data are not plotted, showing the background of the plot. Major commercial inbred name prefixes: LH (Holden's Foundation Seeds, now owned by Bayer), DK (DeKalb Genetics Corporation, now owned by Bayer), PH (Pioneer Hi-Bred International, now owned by Corteva). For full descriptions of inbreds, see the Germplasm Resource Information Network database or (Mazaheri et al., 2019).

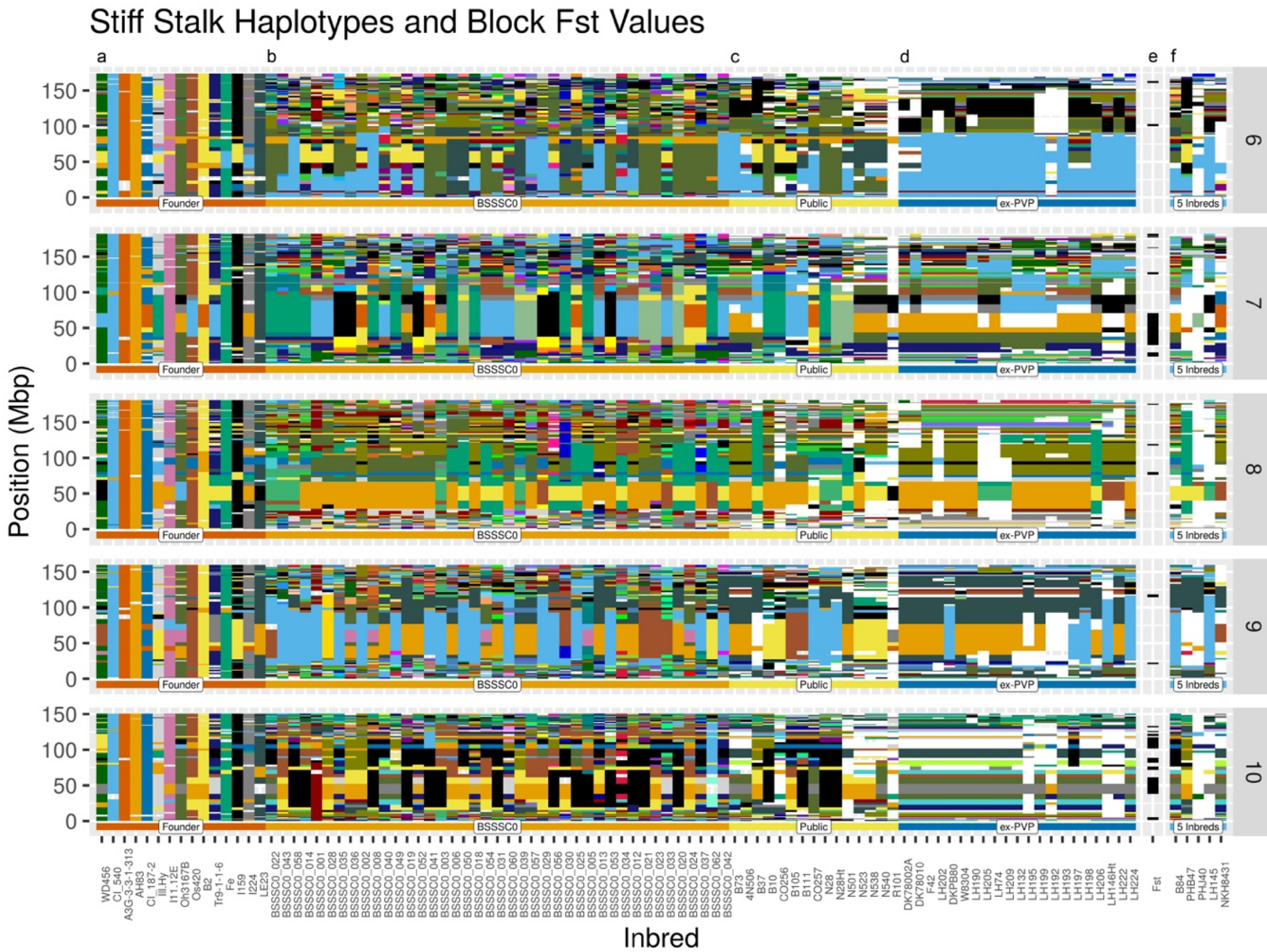

Supplemental Figure S6. Stiff Stalk haplotype and block Fst values for chromosomes six through ten. (a,b) 960 SNP window haplotype blocks for founder inbreds (a) and unselected BSSSC0 inbreds (b) for chromosomes six through ten. (c,d) Conserved BSSS haplotypes for public releases from the BSSS populations (c) and highly related ex-PVP inbreds (d). Haplotypes not found in the founders or BSSSC0 lines are plotted in white. (e) Black boxes indicate haplotypes with binned maximum Fst values in the top 10th percentile of genome-wide binned Fst values. Fst was calculated between the unselected lines, composed of the Founder and BSSSC0 inbreds, and selected lines, composed of the Public and ex-PVP inbreds. (f) Founder and BSSSC0 haplotypes present in the five assembled inbreds. Haplotypes with missing data are not plotted, showing the background of the plot. Major commercial inbred name prefixes: LH (Holden's Foundation Seeds, now owned by Bayer), DK (DeKalb Genetics Corporation, now owned by Bayer), PH (Pioneer Hi-Bred International, now owned by Corteva). For full descriptions of inbreds, see the Germplasm Resource Information Network database or (Mazaheri et al., 2019).

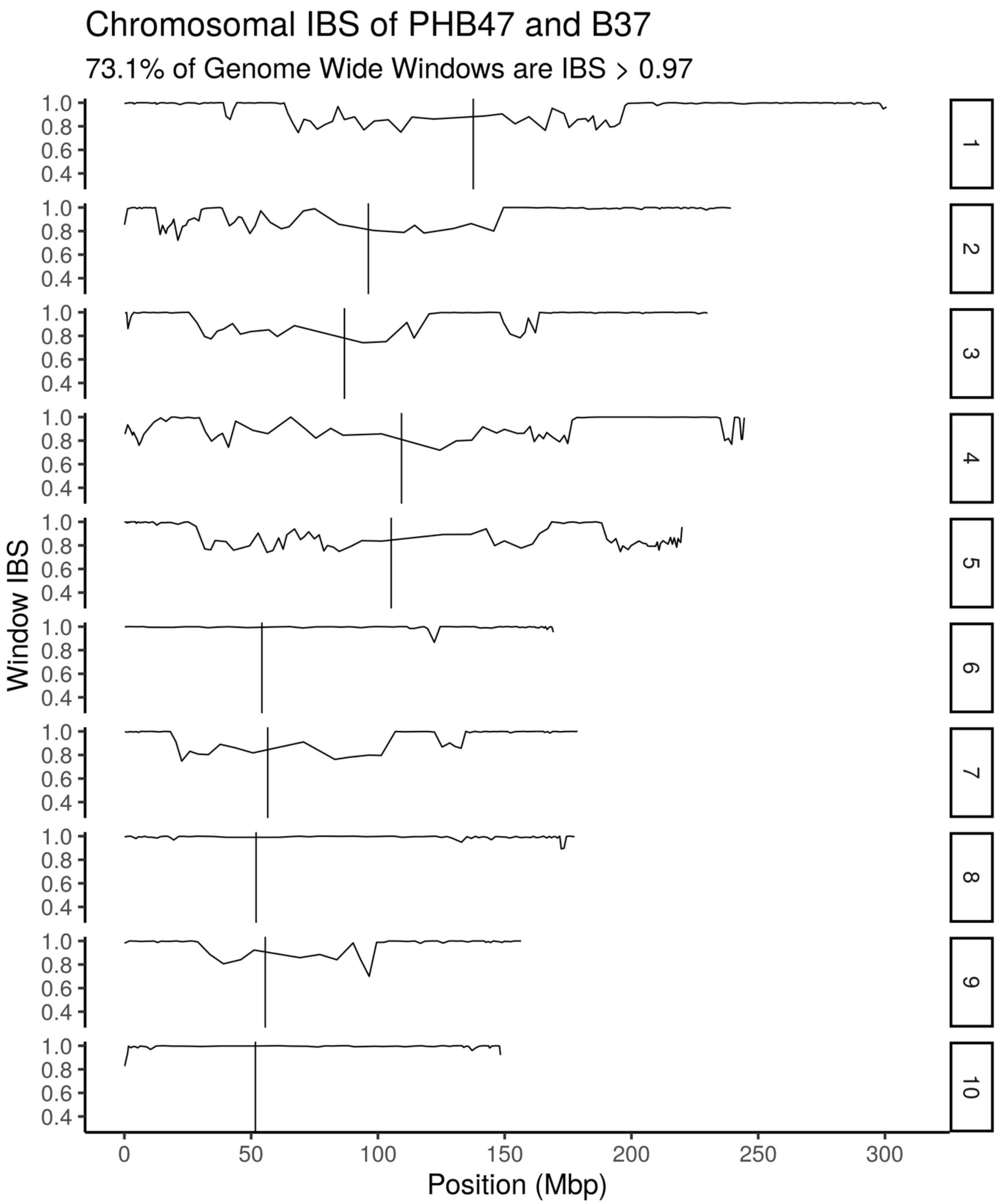

Supplemental Figure S7. Chromosomal IBS of PHB47 and B37. Haplotype window mean identity by state values between PHB47 and B37 are plotted for each of the ten chromosomes, with vertical lines representing the approximate centromere position.

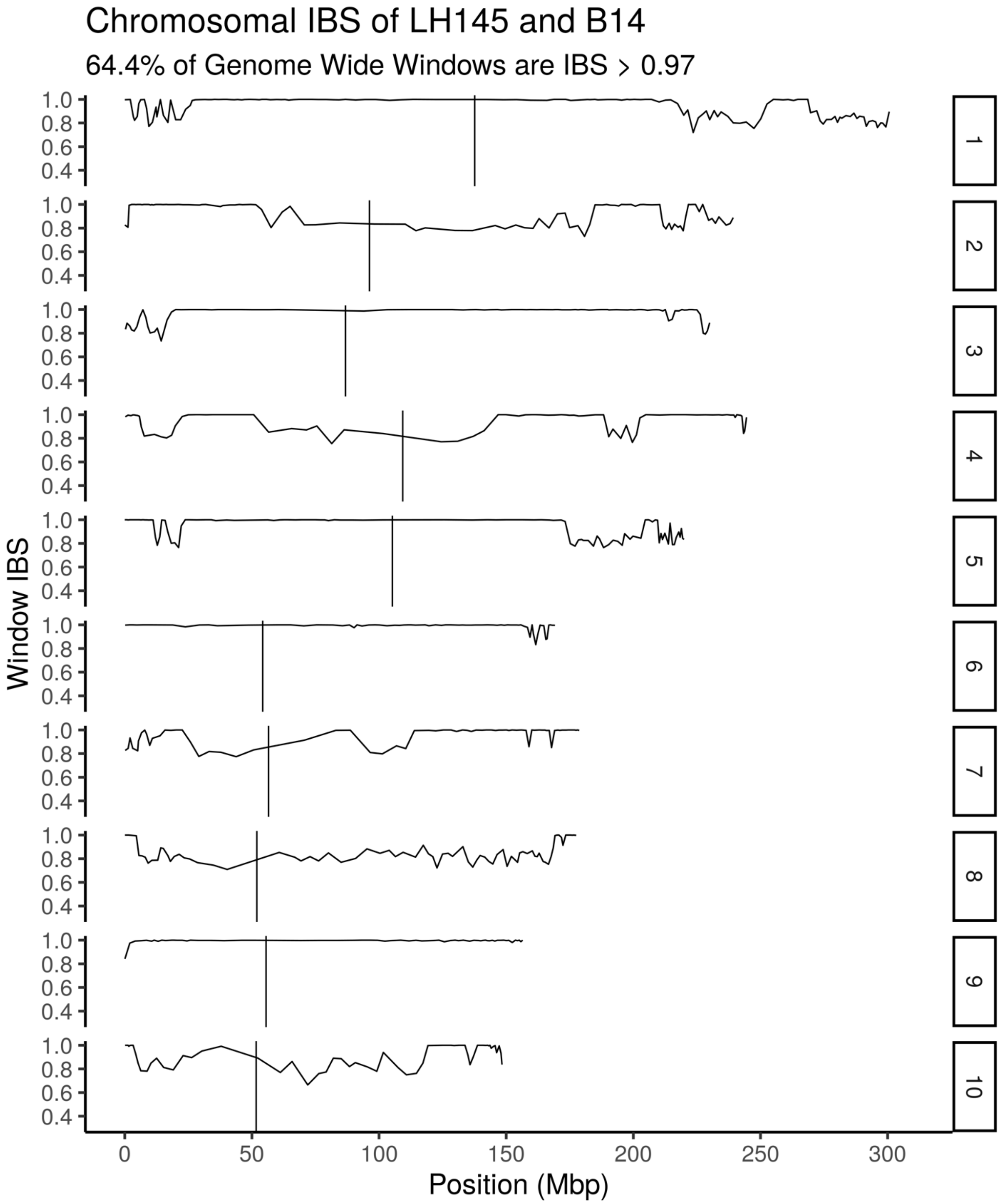

Supplemental Figure S8. Chromosomal IBS of LH145 and B14. Haplotype window mean identity by state values between LH145 and B14 are plotted for each of the ten chromosomes, with vertical lines representing the approximate centromere position.

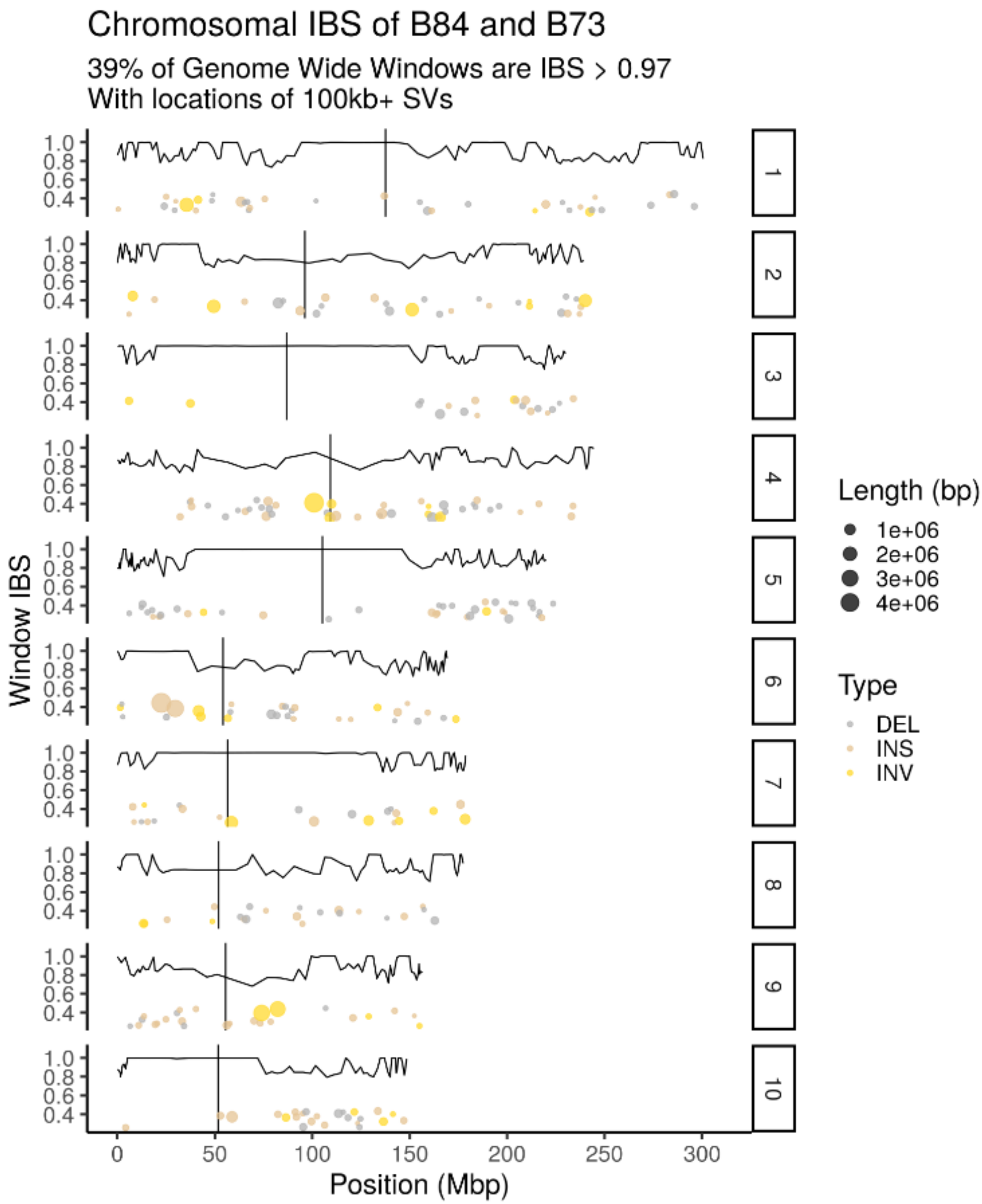

Supplemental Figure S9. Chromosomal IBS of B84 and B73. Haplotype window mean identity by state values between B84 and B73 are plotted for each of the ten chromosomes, with vertical lines representing the approximate centromere position. Locations and lengths of deletions (DEL), insertions (INS) and inversions (INV) are noted as bubbles below the line graph.

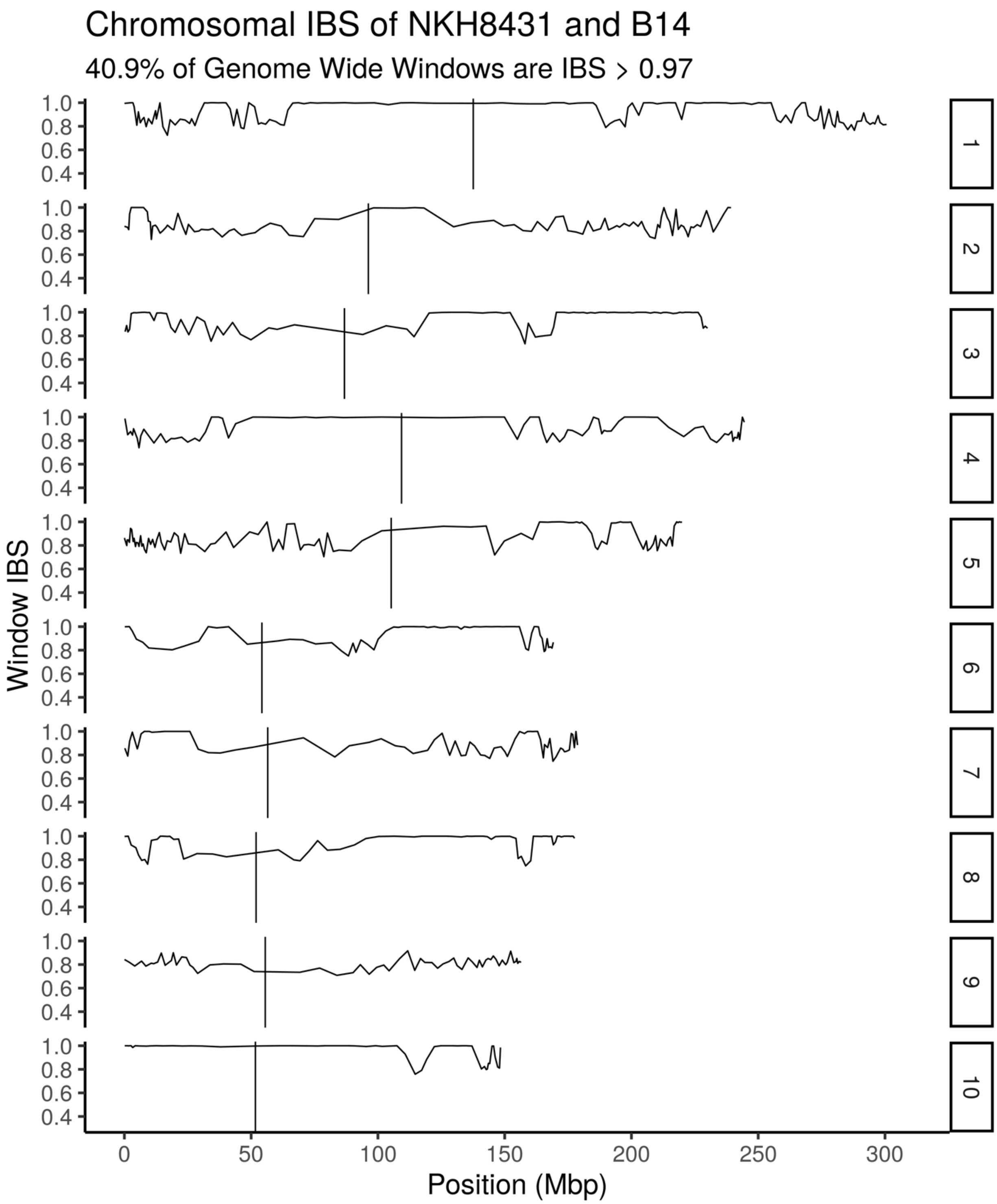

Supplemental Figure S10. Chromosomal IBS of NKH8431 and B14. Haplotype window mean identity by state values between NKH8431 and B14 are plotted for each of the ten chromosomes, with vertical lines representing the approximate centromere position.

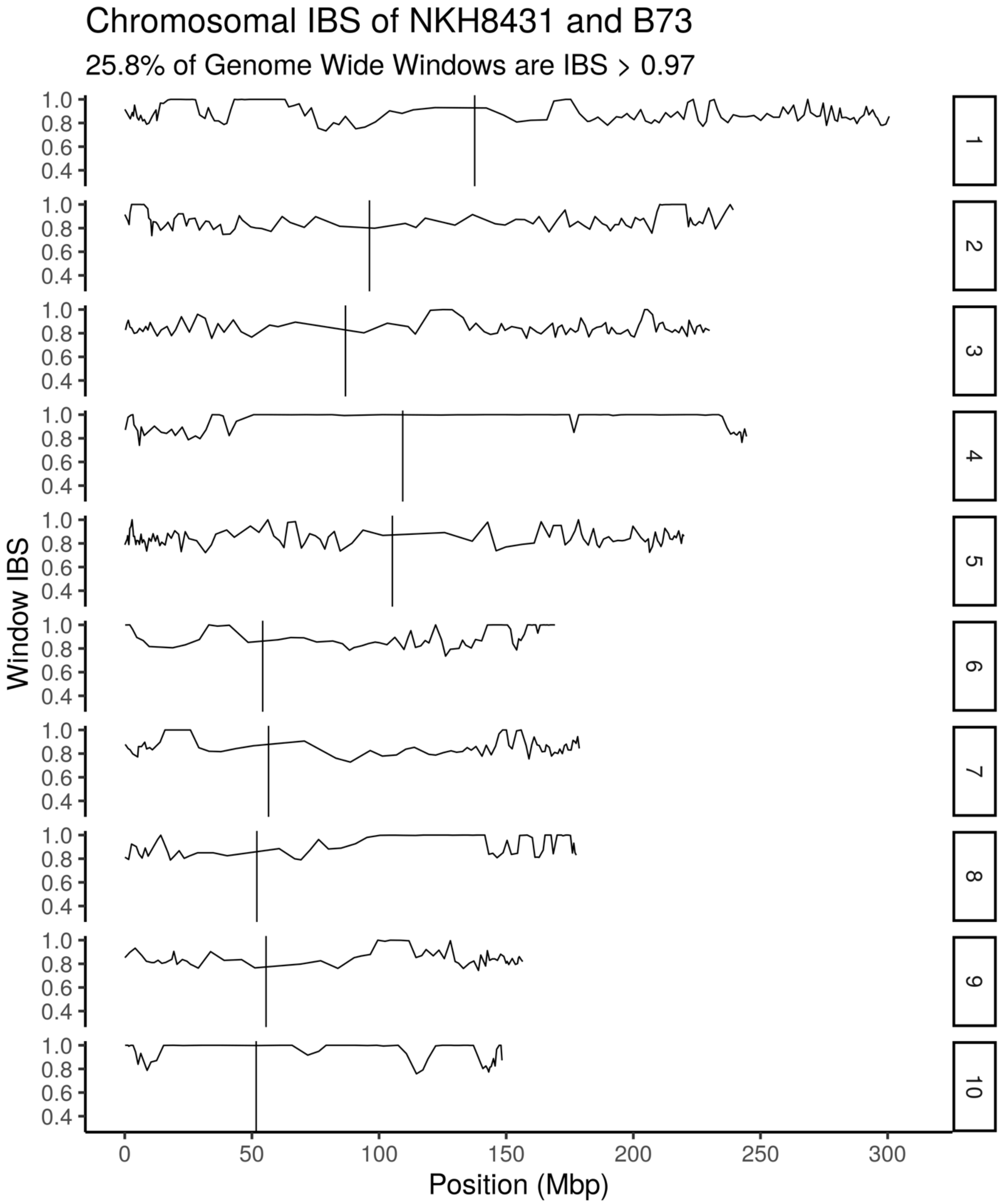

Supplemental Figure S11. Chromosomal IBS of NKH8431 and B73. Haplotype window mean identity by state values between NKH8431 and B73 are plotted for each of the ten chromosomes, with vertical lines representing the approximate centromere position.

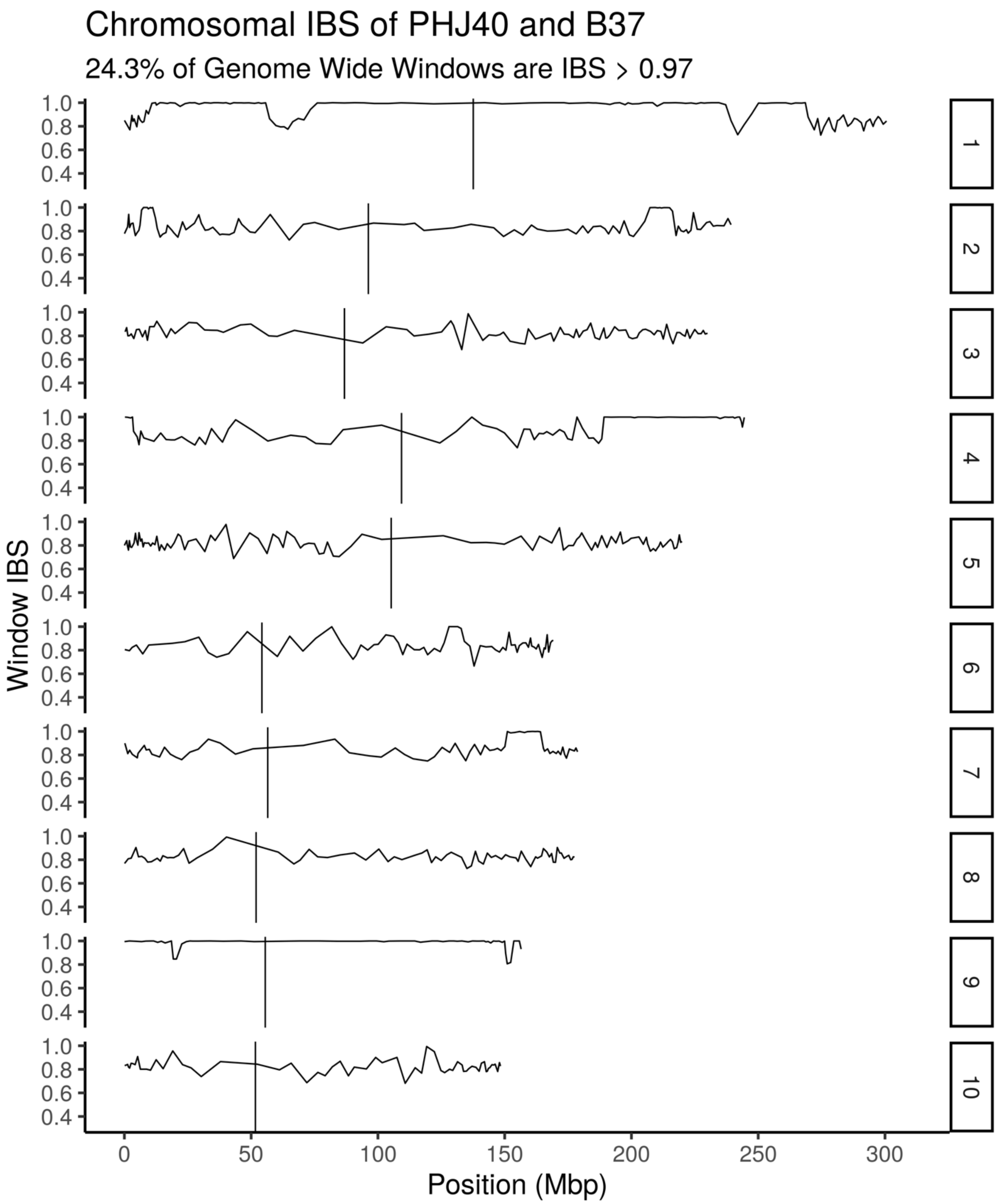

Supplemental Figure S12. Chromosomal IBS of PHJ40 and B37. Haplotype window mean identity by state values between PHJ40 and B37 are plotted for each of the ten chromosomes, with vertical lines representing the approximate centromere position.

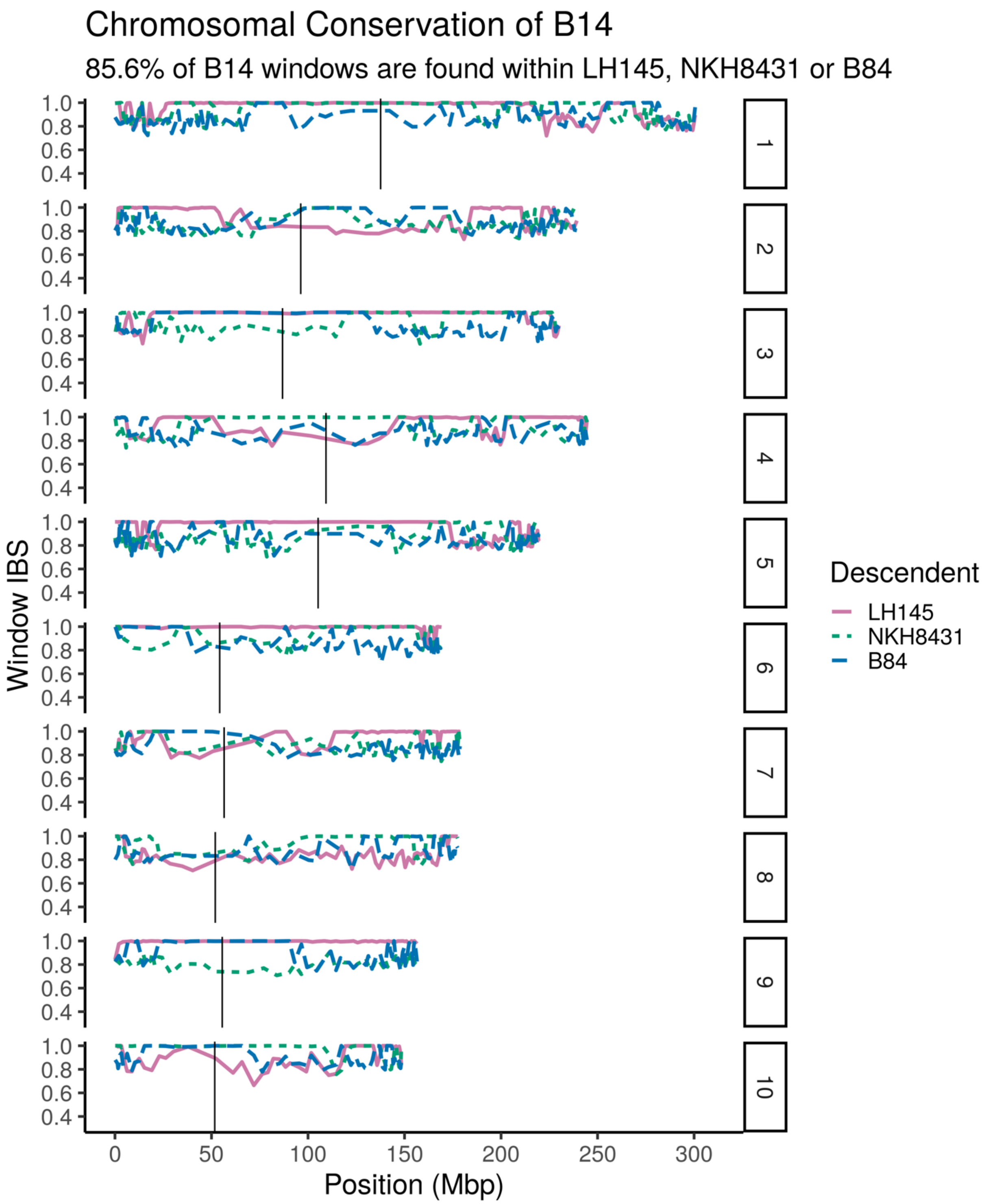

Supplemental Figure S13. Chromosomal conservation of B14. Haplotype window mean identity by state values between B14 and its descendants, LH145 and NKH8431, or related inbred B84 are plotted for each of the ten chromosomes, with vertical lines representing the approximate centromere position.

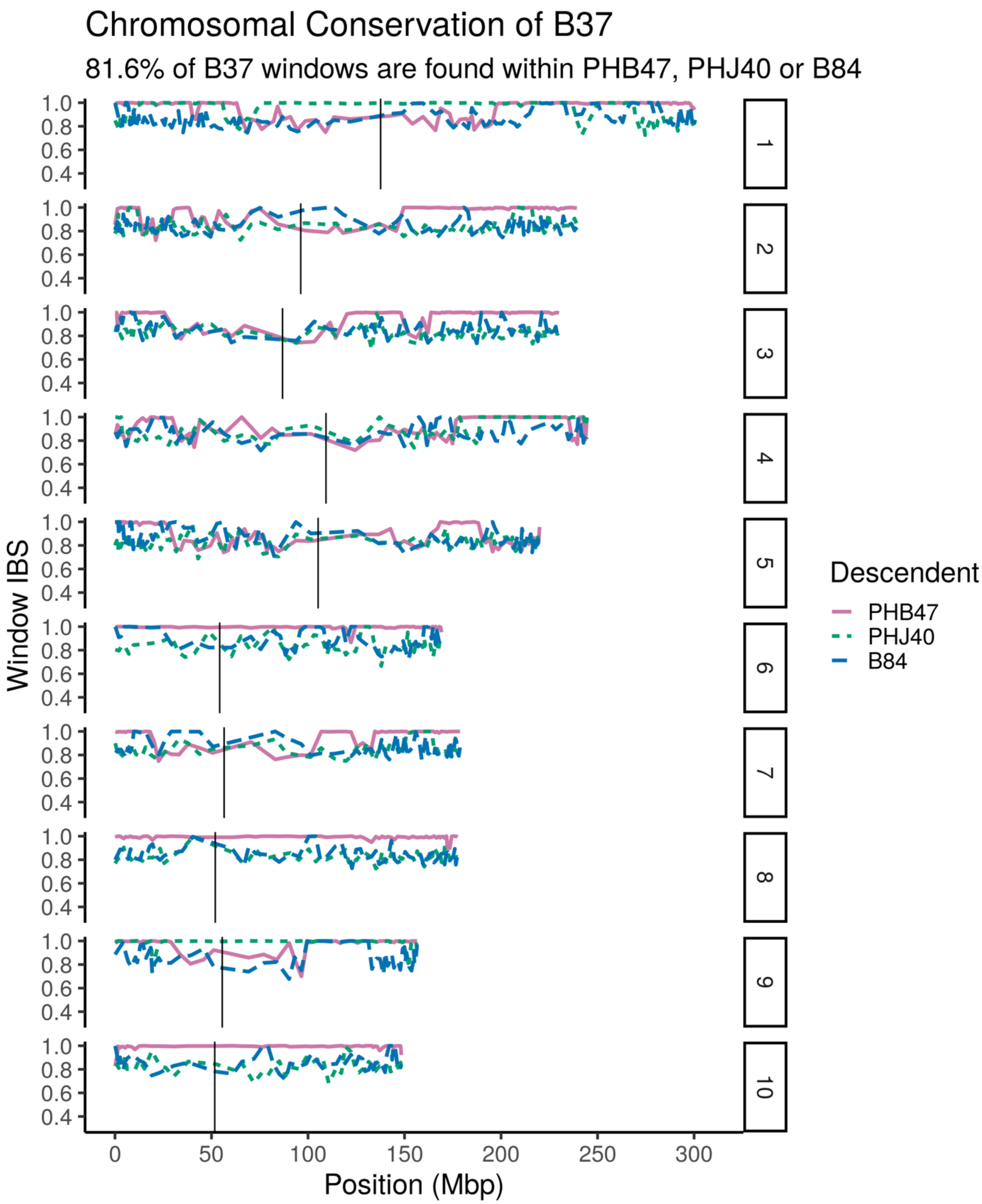

Supplemental Figure S14. Chromosomal conservation of B37. Haplotype window mean identity by state values between B37 and its descendants, PHB47 and PHJ40, or related inbred B84 are plotted for each of the ten chromosomes, with vertical lines representing the approximate centromere position.

Supplemental Table Titles

Supplemental Table S1. Construction of the scaffold assemblies of five Stiff Stalk inbred genomes.

Supplemental Table S2. Whole genome sequencing read mapping statistics for six Stiff Stalk inbreds genome assemblies.

Supplemental Table S3. RNA-Seq read mapping statistics for six Stiff Stalk inbred genome assemblies.

Supplemental Table S4. BUSCO metrics for 6 Stiff Stalk genome assemblies and annotations.

Supplemental Table S5. Summary of the LTR Assembly Index for six Stiff Stalk inbred genomes.

Supplemental Table S6. Percent of genome assembly annotated as a repetitive sequence.

Supplemental Table S7. Pan-orthologous group and protein metrics for six Stiff Stalk inbreds.

Supplemental Table S8. Proportion of high confidence representative coding sequence present in each assembly.

Supplemental Table S9. Pan-gene groupings for six Stiff Stalk inbreds.

Supplemental Table S10. Pan-gene groupings for six Stiff Stalk inbreds, PH207, and Mo17.

Supplemental Table S11. Summary of MCscanX collinear blocks for six Stiff Stalk inbred genomes.

Supplemental Table S12. Disease resistance genes in the predicted proteomes of six maize Stiff Stalks, *Sorghum bicolor*, and *Arabidopsis thaliana*.

Supplemental Table S13. Duplicated DRAGO2 syntelogs on chromosome 1.

Supplemental Table S14. Genome wide window Fst values and haplotype block colors.

References

Clark, R.M., S. Tavaré, and J. Doebley. 2005. Estimating a nucleotide substitution rate for maize from polymorphism at a major domestication locus. Mol. Biol. Evol. 22:2304–2312

Conway, J.R., A. Lex, and N. Gehlenborg. 2017. UpSetR: an R package for the visualization of intersecting sets and their properties. Bioinformatics 33:2938–2940

Mazaheri, M., M. Heckwolf, B. Vaillancourt, J.L. Gage, B. Burdo, S. Heckwolf, K. Barry, A. Lipzen, C.B. Ribeiro, T.J.Y. Kono, H.F. Kaeppler, E.P. Spalding, C.N. Hirsch, C. Robin Buell, N. de Leon, and S.M. Kaeppler. 2019. Genome-wide association analysis of stalk biomass and anatomical traits in maize. BMC Plant Biol. 19:45
